# CD147-high extracellular vesicles promote gastric cancer metastasis via VEGF/AKT/eNOS and AKT/mTOR pathways

**DOI:** 10.1038/s41389-025-00564-3

**Published:** 2025-06-20

**Authors:** Chen-li Zhang, Chan-yuan Zhao, Jia-ming Dong, Cun-pu Du, Bin-sheng Wang, Chen-yu Wang, Wei Liu, Yu-ping Wang, Xiao-yu Zhang, Quan Zhou, Wei Cai, Yun Dang, Li-na Shang, Ai-jun Yang, Min Wang, Min Li

**Affiliations:** 1 Institute of Pathology, School of Basic Medical Sciences, Lanzhou University, Lanzhou, China; 2 The Forensic Identification Unit of Lanzhou University, Lanzhou, China; 3 First Hospital of Lanzhou University, Lanzhou, China; 4 Department of Pathology, Gansu Provincial Hospital, Lanzhou, China; 5 Gansu Provincial Maternity and Child-care Hospital/Gansu Provincial Central Hospital, Lanzhou, China; 6 Medical College of Northwest Minzu University, Lanzhou, China; 7 Experimental Teaching Center of Basic Medicine, School of Basic Medical Sciences, Lanzhou University, Lanzhou, China; 8 Key Laboratory of Preclinical Study for New Drugs of Gansu Province, Lanzhou University, Lanzhou, China

**Keywords:** Tumour biomarkers, Metastasis

## Abstract

Extracellular vesicles (EVs) play a pivotal role in intercellular communication and are closely linked to cancer progression and metastasis. Our previous studies have shown that gastric cancer cell-derived EVs can promote tumor metastasis by increasing the permeability of the endothelial barrier. However, it remains unclear which effector molecule in the EV structure is the key factor of EV-mediated tumor metastasis and the underlying molecular mechanism. In this study, we found that CD147 is a key molecule highly expressed in gastric cancer-derived EVs and confirmed the role of CD147-high EVs from gastric cancer cells in promoting endothelial dysfunction and tumor metastasis. Our results showed that CD147-high EVs activated the VEGF/AKT/eNOS/NO and AKT/mTOR/p70S6K signaling pathways, leading to endothelial cytoskeletal reorganization and internalization of VE-cadherin, which significantly compromised endothelial barrier integrity, increased vascular leakage, enhanced transendothelial migration of tumor cell, and promoted the formation of metastatic tumors. Furthermore, detection of CD147 levels in gastric cancer tissues and plasma EVs indicated that high CD147 expression was associated with advanced tumor stage, poor prognosis, and reduced survival. Our findings suggest that CD147-high EVs are critical mediators of tumor-endothelial interactions and potential diagnostic and prognostic biomarkers for gastric cancer. Their potential as therapeutic targets for gastric cancer is underscored.

**Figure:**
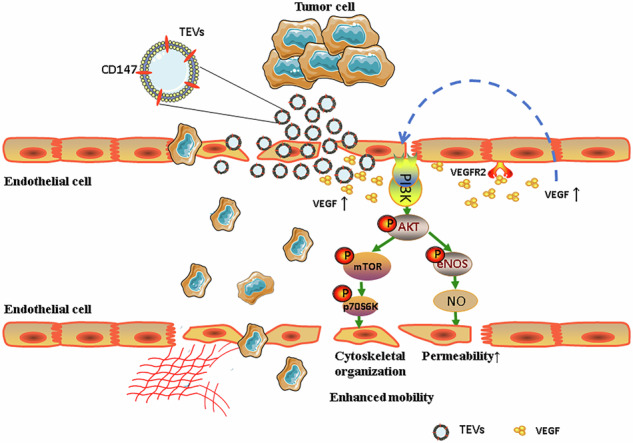
This figure illustrates the proposed mechanism by which CD147-high gcEVs promote tumor metastasis. CD147-high EVs are released from gastric cancer cells and interact with endothelial cells in the tumor microenvironment. Upon uptake by endothelial cells, CD147-high gcEVs activate the key signaling pathways, including the VEGF/AKT/eNOS/NO and AKT/mTOR/p70S6K pathway, which collectively facilitate the metastatic potential of gastric cancer cells by promoting endothelial cell dysfunction and increasing vascular permeability.

This figure illustrates the proposed mechanism by which CD147-high gcEVs promote tumor metastasis. CD147-high EVs are released from gastric cancer cells and interact with endothelial cells in the tumor microenvironment. Upon uptake by endothelial cells, CD147-high gcEVs activate the key signaling pathways, including the VEGF/AKT/eNOS/NO and AKT/mTOR/p70S6K pathway, which collectively facilitate the metastatic potential of gastric cancer cells by promoting endothelial cell dysfunction and increasing vascular permeability.

## Introduction

Gastric cancer (GC) is the fifth most common malignancy and the fourth most deadly globally [1]. While GC screening reduces mortality in East Asia, most global populations face a poor prognosis from delayed diagnosis and metastasis [2, 3]. Addressing these challenges requires a deeper understanding of the molecular mechanisms driving GC metastasis, as well as the identification of sensitive and specific non-invasive biomarkers.

Extracellular vesicles (EVs) are microvesicles released by the donor cells and can be uptaken by the recipient cells, which are considered to be an important intercellular mediator in the tumor microenvironment (TME) [4]. Increasing studies have shown that tumor cell-derived EVs (TEVs) are involved in biological processes such as proliferation, angiogenesis, metastasis, and drug resistance in malignant neoplasms [5–8]. We have previously found that GC-derived EVs (gcEVs) could promote tumor metastasis by increasing the permeability of the endothelial barriers [9]. However, the specific components within EVs that drive these pro-metastatic effects remain unclear. Protein from the cell membrane and cytoplasm in the cargo carried by EVs reflects their biogenesis and functional roles, making them potential candidates for biomarkers and therapeutic targets [10–13]. This raises the possibility that TEVs may exert their pro-metastatic effects through specific membrane proteins.

CD147, also known as Basigin or extracellular matrix metalloproteinase inducer (EMMPRIN), which is a member of the immunoglobulin superfamily. CD147 is abundant in many types of malignant cells and stromal cells [14], and it interacts with several molecules to form complexes, affecting the proliferation, adhesion, invasion, metastasis and energy metabolism of tumor cells via different pathways [15–19]. We identified that CD147 was highly expressed in gcEVs through proteomic analysis. This led us to hypothesize that CD147-high gcEVs regulate endothelial cell function, participate in the formation of pre-metastatic microenvironment, and promote hematogenous metastasis of GC.

In this study, we investigated the mechanisms by which gcEVs exhibiting elevated CD147 expression modulate endothelial cell function, leading to increased endothelial barrier permeability and promotion of GC metastasis. In addition, we evaluated the clinical significance of plasma CD147-positive EVs by correlating their positivity rates with clinicopathological features and patient prognosis. Our objective was to identify a novel biomarker for the early detection and prognostication of GC and to explore potential therapeutic strategies for inhibiting metastatic progression.

## Materials and methods

### Cell culture

Human umbilical vein endothelial cells (HUVECs) and GC cell lines (HGC-27, AGS) were obtained from the Chinese Academy of Sciences Cell Bank (CCTCC, Shanghai, China). The identity of these cell lines was authenticated using short tandem repeat profiling, and they were tested for mycoplasma contamination prior to use. HGC-27 cells were cultured in RPMI 1640 medium supplemented with 10% fetal bovine serum (FBS; Gibco, Thermo Fisher Scientific, Waltham, MA, USA) and 100 U/mL penicillin/streptomycin (HyClone, USA) at 37 °C in a humidified incubator with 5% CO_2_. HUVEC and AGS cells were cultured in DMEM F12 medium (Gibco) under similar conditions.

### Isolation of gcEVs

AGS and HGC-27 cells were cultured to 80–90% confluence, washed twice with phosphate-buffered saline (PBS) (HyClone, USA), and incubated in serum-free medium at 37 °C with 5% CO₂ for 48 h. The conditioned media were collected and centrifuged at 300 × *g* for 10 min at room temperature (RT) to remove cells, followed by centrifugation at 3000 × *g* for 15 min at RT to eliminate cellular debris. The supernatants were subsequently ultracentrifuged twice at 100,000 × *g* for 60 min at 4 °C to obtain the gcEVs, which were resuspended in PBS [20].

### Research cohort

The GC tissues and adjacent non-tumor tissues from 53 GC patients, and plasma samples (5 ml, sodium citrate as anticoagulant) from 67 GC patients and 34 healthy volunteers were collected at the First Hospital of Lanzhou University. None of the patients had received preoperative chemoradiotherapy, immunotherapy, or other treatments. This study was approved by the Ethics Committee of the School of Basic Medical Sciences, Lanzhou University (jcyxy20200324).

### Analysis of EV structural components

Trypsin was used to remove EV surface membrane proteins. EV suspension was treated with 10% trypsin (containing EDTA, final concentration 2%) at 37 °C for 60 min, centrifuged at 20,000 × *g* for 60 min at 4 °C, and the pellet was resuspended in PBS. Repeat the process once.

Sodium dodecyl sulfate (SDS) was used to remove EV surface phospholipids. EV suspension was incubated with 10% SDS (final concentration 2%) and 1% phenylmethylsulfonyl fluoride on ice for 30 min. The SDS-Out reagent (Thermo Fisher Scientific, USA) was added at a 1:20 ratio, and the mixture was vortexed and incubated on ice for 20 min. The suspension was centrifuged at 10,000 × *g* for 10 min at 4 °C, and the supernatant was collected for further analysis [21].

### Proteomics analysis

Protein samples from HGC-27 cells (biological replicates C1, C2, and C3) and HGC-27-derived EVs (E1, E2, and E3) were subjected to label-free quantitative proteomic analysis by Jingjie PTM BioLab (Hangzhou, China).

Secondary mass spectrometry data were analyzed using MaxQuant (version 1.6.6.0) for protein identification and quantification. Gene Ontology (GO) annotation of the protein was primarily derived from the UniProt-GOA database (available at http://www.ebi.ac.uk/GOA/). Additionally, protein annotations corresponding to the Kyoto Encyclopedia of Genes and Genomes (KEGG) database were obtained using the KEGG Automatic Annotation Server. The KEGG Mapper tool was then employed to map the KEGG Orthology numbers to specific biological pathways. Data visualization was performed using the ggplot2 package (version 3.3.3) in R.

### Confocal laser scanning microscopy

#### Observation of endothelial cell uptake of EVs using live cell imaging

HUVECs were co-incubated with PKH26-labeled EVs for 12 h in a live cell workstation, monitored continuously using a laser confocal microscope (LSM 900, Zeiss, Germany).

#### FITC-phalloidin staining

HUVECs were incubated with different EVs and PBS, MK2206 (20 μM), SC79 (20 μM), or Rapamycin (100 nM) were added for 24 h, followed by fixation in 4% paraformaldehyde for 10 min. The cells were permeabilized with 0.5% Triton X-100 for 5 min and incubated with 5 μg/mL FITC-phalloidin (Sigma, USA) for 1 h to stain filamentous actin (F-actin). The cells were washed with PBS and sealed with mounting medium containing 4′,6-diamidino-2-phenylindole (DAPI). The actin cytoskeleton was visualized under a confocal microscope.

#### Assessment of VE-cadherin localization

After different treatments for 24 h, HUVECs were incubated overnight at 4 °C with anti-VE-cadherin antibody (1:200, Gene Tex, GXT633705). Goat anti-mouse IgG (1:200, Abcam) conjugated with Alexa Fluor 594 was followed by incubation for 1 h at RT. Cells were washed with PBS, mounted with DAPI-containing medium, and observed under a confocal microscope to evaluate VE-cadherin localization.

### Transendothelial electrical resistance (TEER) assay

TEER assay was performed using a Millicell ERS-2 Voltammeter (Merck KGaA, Darmstadt, Germany) to assess endothelial barrier integrity. HUVECs were seeded in the upper chamber of Transwell inserts (0.4 μm pore size, Corning, NY, USA) and cultured until confluent. Then, gcEVs or PBS or recombinant protein CD147 (2 μg, Novoprotein, China) were added to the upper chamber and co-incubated at 37 °C in 5% CO_2_ for 24 h. TEER was measured at different time points using a Millicell® ERS-2 Resistance Meter and calculated per unit area resistance according to the manufacturer’s instructions. Three replicates of wells were set up for each experiment.

### FITC-dextran permeability assay

HUVECs were seeded in the upper chamber of Transwell inserts (0.4 μm pore size) and allowed to reach confluence. After gcEVs or PBS or recombinant protein CD147 were added to the upper chamber, the media in both upper and lower chambers were replaced with phenol-red-free and serum-free media. FITC-dextran (100 μg/mL, Sigma, USA) was added to the upper chamber and incubated for 4 h. The lower chamber medium was then collected, mixed, and transferred to a black 96-well plate (150 μL/well). Fluorescence intensity was measured using a microplate reader (Molecular Devices, Sunnyvale, CA) at an excitation wavelength of 485 nm and an emission wavelength of 535 nm.

### Evans Blue extravasation assay

The Evans Blue extravasation assay was employed to measure EV-induced pulmonary vascular permeability in mice [9, 22]. Six-week-old male BALB/c-Nude mice (Jiangsu GemPharmatech Co., Ltd, China) were randomly divided into three groups, each receiving the following via tail vein injection: (1) shNC-EVs derived from HGC-27 cells (2 × 10^6^ EVs/mouse); (2) shCD147-EVs derived from HGC-27 cells (2 × 10^6^ EVs/mouse); and (3) an equal volume of PBS (*n* = 3 per group). After 24 h, each mouse received a tail vein injection of 200 μL of 0.5% Evans Blue dye (Solarbio Science, Technology Co., Ltd., Beijing, China). Thirty minutes after dye injection, the mice were euthanized, and lung tissues were harvested for further analysis.

The right lung was collected, weighed, and incubated with Hi-Di™ Formamide (Thermo Scientific, USA) at 0.02 g/150 μL in a 56 °C water bath for 24 h. The samples were then centrifuged at 1500 × *g* for 10 min, and the supernatant was collected for measurement of Evans Blue dye concentration at 620 nm using a microplate reader (Bio-Rad Laboratories, Hercules, CA, USA). The left lung was fixed in 4% paraformaldehyde (Solarbio), embedded in paraffin, sectioned, and stained with hematoxylin and eosin for histological analysis.

### Transendothelial migration assay

HUVECs were seeded in the upper chamber of Transwell inserts (8 μm pore size, Corning, NY, USA) and allowed to reach full confluence. In all, 1 × 10^5^ PKH-26-labeled HGC-27 cells were added to the upper chamber along with different EVs in serum-free media, while the lower chamber contained media with 15% FBS. After incubation at 37 °C in 5% CO_2_ for 24 h, the number of cells migrating to the lower surface of the Transwell membrane was counted using fluorescence microscopy.

### Mouse model

Six-week-old male NTG mice (Speifer, Beijing, Laboratory Animal Technology Co., Ltd) were used to construct the metastatic model. Briefly, the mice were randomly divided into 3 groups and administered, respectively, the following treatments via tail vein injection: (1) 2 × 10^6^ HGC-27 cells; (2) 2 × 10^6^ HGC-27 cells mixed with 1 × 10^6^ shNC-derived EVs; (3) 2 × 10^6^ HGC-27 cells mixed with 1 × 10^6^ shCD147-derived EVs. The injection volume was normalized at 200 μL per mouse. Following the initial injection, shNC-EVs or shCD147-EVs or PBS were administered at the same dose every 3 days for 30 days. Mice were subsequently euthanized under anesthesia, and blood samples were collected immediately (0.32% sodium citrate as anticoagulant). Lungs and liver were also collected and served as the outcome measure. All animal procedures were conducted in accordance with the guidelines of the Institutional Animal Care and Use Committee (IACUC) of Lanzhou University (Lzujcyxy20240102).

### Statistical analysis

All data were statistically analyzed using R software (version 3.6.1) and GraphPad Prism (version 9, GraphPad Software, La Jolla, CA). Data were presented as mean ± standard deviation (mean ± SD). The Shapiro–Wilk test was used to assess the normality of the data distribution. Levene’s test was employed to verify the homogeneity of variances. For normally distributed data, comparisons were made using Student’s unpaired two-tailed *t* test and one-way analysis of variance followed by Bonferroni’s post hoc test for multiple comparisons. For non-normally distributed data, the Mann–Whitney *U*-test and Spearman’s rank correlation analysis were used. A *p* value < 0.05 was considered statistically significant.

## Results

### Protein components of EVs influence endothelial barrier permeability

**Fig. 1:**
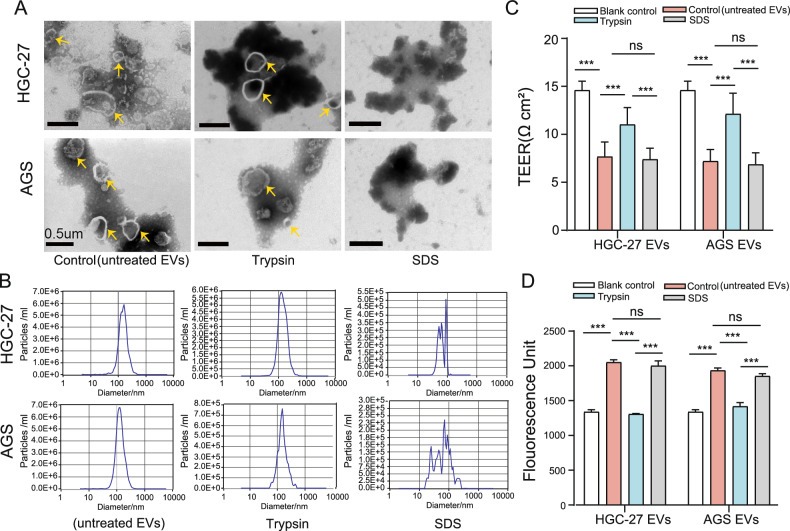
Protein components in gcEVs affected endothelial cell permeability. **A** Phosphotungstic acid-negative staining TEM images of gcEVs treated with trypsin or SDS (bar = 0.5 μm, yellow arrow: EVs). **B** Nanoparticle tracking analysis (NTA) of EV size distribution. EVs treated with different conditions were co-incubated with HUVECs of confluent monolayers for 24 h. Untreated EVs were used as the Control. **C** Transendothelial electrical resistance (TEER) was measured (*n* = 9, one-way ANOVA). **D** FITC-dextran permeability assay to detect the fluorescence intensity of FITC-dextran diffusing to the lower chamber (*n* = 5, one-way ANOVA). Untreated EVs were used as the Control, and untreated HUVECs as the Blank control. ns, not statistically significant, ***p < 0.001.

First, we extracted EVs derived from HGC-27 and AGS cells (gcEVs), which were consistent with EV characterization (Fig. S1) according to previous studies [19]. Subsequently, gcEVs were treated with 10% trypsin to remove membrane proteins while preserving the phospholipid structure of the cell membrane. Nanoparticle tracking analysis (NTA) confirmed that enzymatic treatment did not significantly alter particle size distribution. However, treatment with 2% SDS completely disrupted the phospholipid bilayer, leading to significant changes in particle size (Fig. 1A, B).

Next, we assessed the effects of different components of gcEVs on the endothelial permeability by TEER and FITC-dextran permeability assays. The transendothelial resistance value of gcEVs in the trypsin-treated group was statistically higher than that in the control(untreated EVs) or SDS-treated group (*p* < 0.001) (Fig. 1C). The higher the resistance value, the lower the permeability. FITC-dextran permeability measurement showed that the fluorescence intensity of the lower chamber in the trypsin-treated group was remarkably weaker than that in the control (untreated EVs) or SDS-treated group over 24 h following the treatment of monolayer endothelial cells (*p* < 0.001) (Fig. 1D). These data indicate that the effect of gcEVs on endothelial permeability is significantly attenuated by removing the protein component of EVs, suggesting that the membrane proteins of EVs play a key role in regulating endothelial barrier function.

### CD147 as one of the major effectors mediating the biological functions of gcEVs

**Fig. 2:**
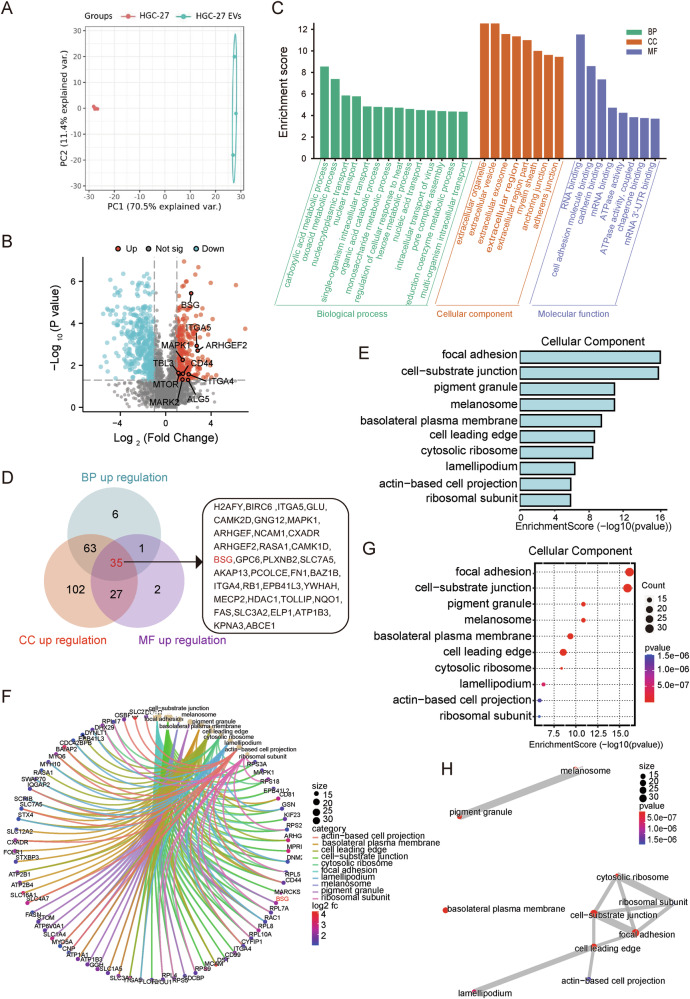
Proteomic analysis of HGC-27 cells and HGC-27-derived EVs. **A** Principal component analysis (PCA) of differentially expressed proteins in HGC-27 cells and HGC-27-derived EVs. **B** The volcano plot visualized the differential expression of identified proteins in HGC-27 cells and HGC-27-derived EVs (red dots indicate significantly upregulated proteins, blue dots represent significantly downregulated proteins, and gray dots indicate non-significant proteins). **C** Gene Ontology (GO) enrichment analysis of the differentially expressed proteins, categorized into biological process (BP), cellular component (CC), and molecular function (MF) (fold change >2, *p* < 0.05). **D** Venn diagram showing the overlapping proteins that are upregulated in the GO analysis across BP, CC, and MF categories (Fold change >2, *p* < 0.05). **E** Bar plot displaying the top 10 enriched CC terms ranked by statistical significance (−log10 (*p* value)). **F** Network map illustrating the relationship between upregulated proteins and their associated CC terms. **G** Bubble plot of the enriched top 10 CC terms, where color intensity represents *p* value and bubble size indicates protein count. **H** Interaction network of top 10 upregulated CC terms. Node size corresponds to the number of enriched proteins (larger circles indicate more proteins). Node color indicates the significance level (−log10 transformed *p* value, darker red indicates greater significance). Gray edges represent functional relationships, with edge width proportional to shared gene counts. The network was constructed using Cytoscape (v3.9.1), with FDR < 0.05 as the significance threshold.

The results of Fig. 1 demonstrate clearly that membrane proteins are the main effector molecules of EVs to increase the permeability of the endothelial barrier. Therefore, we conducted proteomic analysis of the EVs derived from HGC-27 cells to find the main effector protein of EVs. A total of 5001 proteins were identified by mass spectrometry, of which 2185 proteins were able to be quantified (Supplementary Table 1). Principal component analysis (PCA) of the proteins from all samples revealed good clustering of both HGC-27 cells and HGC-27 -derived EVs (PC1 = 70.5%, PC2 = 11.4%), indicating a good repeatability of the experiments (Fig. 2A). Compared with HGC-27 cells, 246 proteins were significantly upregulated and 423 proteins were downregulated in HGC-27-derived EVs. Some of the differentially upregulated proteins have been highlighted in the volcano plot. (Fig. 2B). We performed GO enrichment analysis on differentially expressed proteins in HGC-27 cells and HGC27-derived EVs, revealing the characteristics of these differentially expressed proteins in biological processes (BP), cellular components (CC), and molecular functions (MF) (Fig. 2C).

To further identify specific regulatory proteins in HGC-27-derived EVs, we performed a more in-depth GO analysis of 246 upregulated proteins. The Venn diagram revealed 35 overlapping proteins across the three GO categories of BP, CC and MF, and the *BSG* gene (CD147) was found among both the differentially upregulated proteins and the overlapping sets in all three classes (Fig. 2D). Subsequently, we analyzed the top ten BP terms and associated protein counts, indicating that upregulated proteins were primarily involved in regulating cell morphology, with network analysis confirming that the upregulated *BSG* gene played a role in regulating cell morphology (Fig. S2). Analysis of the top ten CC terms and associated protein counts revealed that upregulated proteins are predominantly involved in focal adhesion and cell-matrix attachment regulation (Fig. 2E, G), with network analysis confirming the role of the *BSG* gene in focal adhesion and cell–matrix attachment regulation (Fig. 2F, H). KEGG pathway enrichment analysis of GO molecular function-upregulated proteins in HGC-27-derived EVs identified the top enriched pathways, including regulation of actin cytoskeleton, alongside pathways related to viral infection and cancer. Notably, network analysis confirmed the involvement of the BSG gene in cytoskeletal regulation, highlighting its potential role in modulating cellular architecture and metastatic processes (Fig. S3).

We subsequently verified the expression of CD147 in gcEVs and found that CD147 was significantly highly expressed in EVs derived from HGC-27 and AGS cells, with fold changes of 12.27 and 15.36, respectively (Fig. S4A). Therefore, we selected CD147 for further investigation.

### CD147-high gcEVs promote tumor cell transendothelial migration and metastasis

**Fig. 3:**
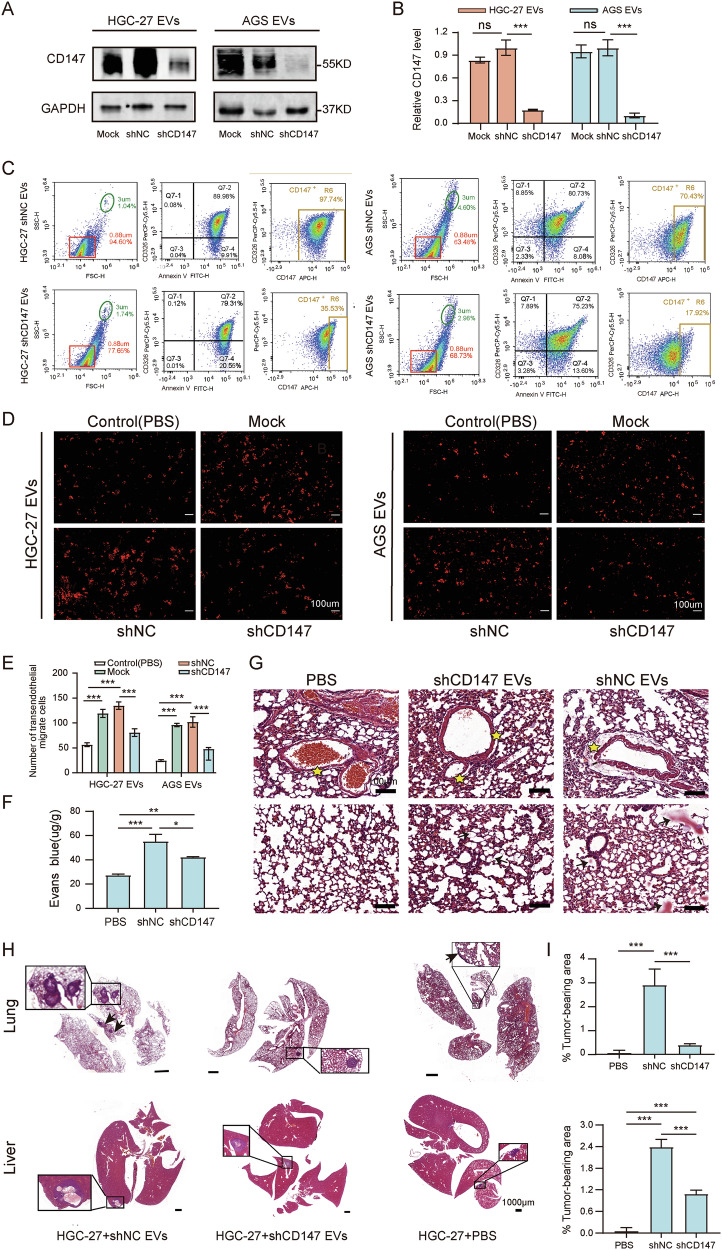
CD147-high gcEVs disrupted the vascular endothelial barrier and promoted tumor cell transendothelial migration and metastasis. **A** Western blot analysis of CD147 levels in EVs. Mock EVs (derived from untreated cells), shNC EVs (derived from cells transfected with non-targeting shRNA), and shCD147 EVs (derived from cells transfected with CD147-targeting shRNA). **B** Quantification of CD147 expression (*n* = 3, one-way ANOVA). **C** Flow cytometry analysis of CD147 levels in EVs from transfected cells. **D** Transendothelial migration assay: HUVECs were cultured to confluence and treated with different groups, followed by co-culture with PKH-26-labeled HGC-27 cells (1 × 10^5^) for 24 h. The number of migrating HGC-27 cells in the lower chamber was quantified using fluorescence microscopy. **E** Statistical analysis of tumor cell transendothelial migration (*n* = 3, one-way ANOVA). **F** Detection of Evans blue dye leakage into lung parenchyma in mice treated with different groups (*n* = 3, one-way ANOVA). **G** Representative H&E-stained lung sections from mice subjected to the three treatments (bar = 100 μm; asterisk: perivascular space, arrow: alveolar exudate). **H** H&E-stained sections of lungs and liver from different treatment groups (bar = 1000 μm; arrow: tumor tissue). **I** Analysis of cancerous regions in lung and liver tissues. *p < 0.05, **p < 0.01, ***p < 0.001.

To investigate the biological effects of CD147-gcEVs, we employed lentiviral transduction to knock down CD147 expression in HGC-27 and AGS cells, with mRNA and protein inhibition efficiencies of over 65%, 71% and 66%, 76%, respectively (Fig. S4B–D). The levels of CD147 in the gcEVs extracted from CD147-knockdown cells were significantly reduced (Fig. 3A–C).

We then completed a transendothelial assay to observe the migration of GC cells. The results displayed that CD147-high gcEVs significantly promoted transendothelial migration of GC cells, whereas CD147-knockdown gcEVs suppressed this effect (*p* < 0.001) (Fig. 3D, E). We found that CD147-high gcEVs significantly increased vascular leakage in mouse lungs through the Evans blue extravasation assays, whereas CD147-knockdown gcEVs reduced this effect (Fig. 3F). Mouse lung histopathology also showed that CD147-high gcEVs increased vascular permeability significantly, manifested by expanded perivascular spaces and alveolar spaces filled with exudative plasma proteins (Fig. 3G). Mouse transfer model confirmed that CD147-high gcEVs significantly increased the area of GC metastases in the lungs and liver of mice, whereas CD147-knockdown gcEVs significantly reduced the formation of metastatic lesions (Fig. 3H, I). These results indicate that CD147-high gcEVs have an effect on increasing endothelial barrier permeability, thus promoting the transendothelial migration and organ metastasis of GC cells.

### CD147-high gcEVs disrupted endothelial barrier integrity by cytoskeletal reorganization and VE-cadherin internalization

To investigate the effect of CD147-high gcEVs on endothelial barrier, the CD147-high or CD147-knockdown gcEVs were incubated with HUVECs at 37 °C for 24 h, and the changes in HUVEC permeability were observed by measuring TEER and dextran permeability through the endothelium.

**Fig. 4:**
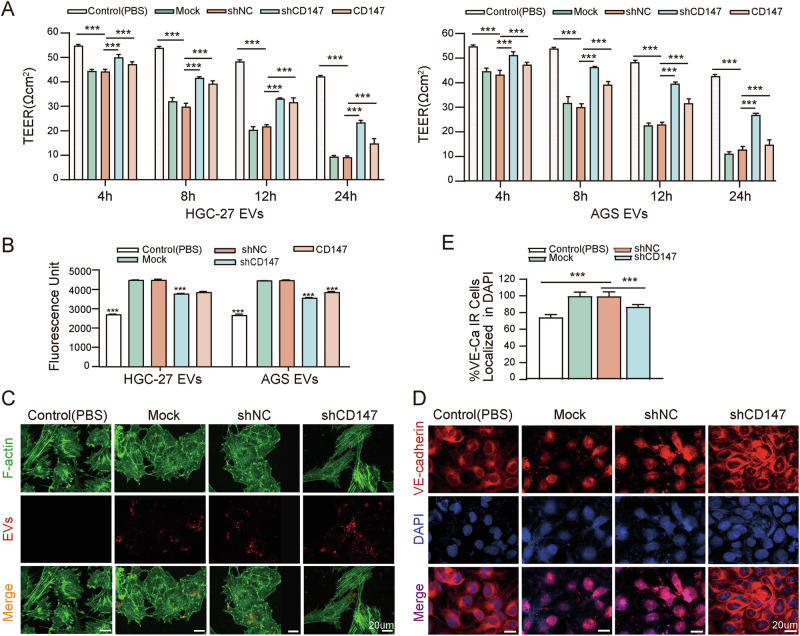
CD147-high gcEVs affected endothelial barrier permeability by inducing cytoskeletal rearrangement and VE-cadherin internalization. **A** Transendothelial electrical resistance (TEER) measurement in permeability of HUVECs treated with Mock EVs, shNC EVs, shCD147 EVs, PBS, or recombinant CD147 protein at different time points (*n* = 3, one-way ANOVA). **B** Permeability assay in confluent HUVECs treated with Mock EVs, shNC EVs, shCD147 EVs, PBS, or recombinant CD147 protein for 24 h; FITC-dextran was added to the upper chamber, and the fluorescence intensity in the lower chamber was measured after 24 h (*n* = 3, one-way ANOVA, vs. shNC). **C** Confocal microscopy images of endothelial cells treated with different gcEVs or PBS for 24 h, showing F-actin distribution (green: F-actin, blue: nucleus, red: gcEVs, bar = 20 μm). **D** Confocal microscopy images displaying VE-cadherin localization in HUVECs treated with different gcEVs or PBS for 24 h (red: VE-cadherin, blue: nucleus, bar = 20 μm). **E** Quantification of the colocalization coefficient of VE-cadherin with the nucleus (*n* = 3, one-way ANOVA). ***p < 0.001.

The results demonstrated that CD147-high gcEVs markedly decreased the monolayer resistance value of endothelial cells and increased dextran leakage through the endothelium. In contrast, CD147-knockdown gcEVs significantly attenuated the endothelial barrier disruption (*p* < 0.001 vs. shNC EVs), with preserved monolayer resistance and reduced dextran permeability. Although CD147 recombinant protein also enhances the permeability of the endothelial barrier, it is not as effective as CD147-high gcEVs (Fig. 4A, B).

We investigated the mechanism of increasing endothelial permeability by CD147-high gcEVs on endothelial permeability from two perspectives. On the one hand, HUVECs were co-incubated with gcEVs and observed for 12 h continuously under a laser confocal microscope. It was found that CD147-high gcEVs were gradually endocytosed by HUVECs, and the number of gcEVs uptaken increased in a time-dependent manner (Fig. S5). At the same time, we assessed the impact of gcEVs on the endothelial cytoskeleton. In the control group, F-actin fibers were organized parallel to the long axis of the endothelial cells. However, treatment with CD147-high gcEVs disrupted this arrangement, leading to diffuse and fragmented F-actin fibers. In contrast, HUVECs treated with CD147-knockdown gcEVs maintained predominantly elongated F-actin fibers, with reduced cytoskeletal deformation (Fig. 4C).

On the other hand, we detected changes in endothelial cell adhesion molecules. VE-cadherin plays crucial functions in adhesion junctions between endothelial cells and in maintaining the vascular integrity and permeability [23]. We found that HUVECs treated with CD147-high gcEVs exhibited significant internalization of VE-cadherin, whereas CD147-knockdown gcEVs markedly inhibited this phenomenon (Fig. 4D, E). These results remind us that endocytosis of CD147-high gcEVs by endothelial cells leads to cytoskeleton rearrangement and cell contraction, reducing intercellular adhesion, and enlarging the intercellular space and endothelial barrier permeability.

### CD147-high gcEVs exert effects via VEGF/AKT/eNOS/NO and AKT/mTOR pathways

To investigate signaling pathways potentially involved in CD147-high gcEV function, we mapped MF-upregulated proteins to KEGG reference pathways using KEGG Mapper. This analysis revealed that these proteins were mapped to the GC pathway and the PI3K-AKT signaling pathway, highlighting key regulators such as mTOR, S6K, AKT, and eNOS (Fig. S6). Given the known roles of these pathways in vascular permeability and tumor progression, we further explored their functional relevance in CD147-high gcEVs.

**Fig. 5:**
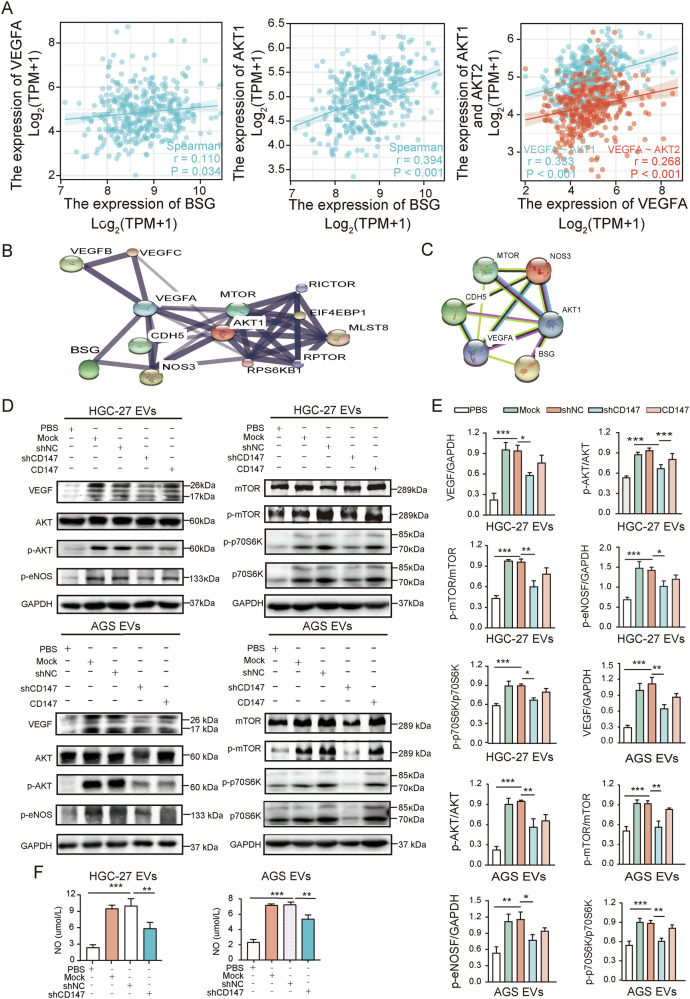
CD147-high gcEVs regulated endothelial cell function via the VEGF/AKT/eNOS/NO and AKT/mTOR pathways. **A** Correlation analysis of BSG (CD147) with VEGFA, and BSG (CD147) with AKT1, as well as VEGFA with AKT1 and AKT2 in GC tissues based on the TCGA database. **B** STITCH database analysis of protein-protein interactions, with line thickness indicating the strength of data support (thicker lines represent stronger reliability). **C** STRING database analysis illustrating the protein–protein interaction network. **D** Western blot analysis of VEGF, p-AKT, AKT, p-eNOS, p-mTOR, mTOR, p-p70S6K, and p70S6K expression in HUVECs treated with different gcEVs, PBS, or recombinant CD147 protein for 24 h, and **E** quantitative analysis (*n* = 3, one-way ANOVA). **F** Measurement of NO levels in the culture supernatant of HUVECs co-incubated with different EVs or PBS for 24 h (*n* = 3, one-way ANOVA). *p < 0.05, **p < 0.01, ***p < 0.001, vs shNC.

To further validate these findings, we analyzed correlations in the TCGA database and found a positive association between BSG (CD147), VEGFA, AKT1, and AKT2 (Fig. 5A). We then evaluated protein–protein interactions using the STITCH and STRING databases, which demonstrated prominent interactions between BSG (CD147) and key signaling molecules, such as VEGF, AKT, eNOS, mTOR, and VE-cadherin (Fig. 5B, C), thereby supporting the involvement of these pathways.

Subsequent experimental validation was performed in HUVECs. Western blot analysis showed that treatment with CD147-high gcEVs markedly increased the expression of VEGF, p-AKT, p-eNOS, p-mTOR, and p-p70S6K and promoted the release of NO. On the contrary, CD147- knockdown gcEVs significantly reduced the expression of these proteins as well as NO release (Fig. 5D–F). The AKT inhibitor MK2206 inhibited the activation of p-AKT, p-eNOS, and p-mTOR proteins and NO release by CD147-high gcEVs, whereas the AKT agonist SC79 reversed the inhibition of these proteins’ expression and NO release by CD147-knockdown gcEVs (Fig. S7A–C). Rapamycin, an mTOR inhibitor, suppressed p-p70S6K activation by CD147-high gcEVs (Fig. S7D, E).

MK2206 and SC79 were further used to validate the effect of the AKT pathway on the endothelial barrier by CD147-high gcEVs. The results suggested that MK2206 reversed the CD147-high gcEVs induced promotion of endothelial cell permeability, tumor cell transendothelial migration, cytoskeletal rearrangement, and internalization of VE-cadherin, whereas SC79 reverted the inhibitory effect of CD147-knockdown gcEVs (Figs. S8 and S9). CD147-high gcEVs induced diffuse and fragmented F-actin fibers, which were restored to a more organized state upon rapamycin treatment. Similarly, VE-cadherin internalization induced by CD147-high gcEVs was markedly reduced in the presence of rapamycin, as evidenced by increased membrane localization of VE-cadherin (Fig. S10).

### CD147 used as a marker for diagnosis and poor prognosis of GC

**Fig. 6:**
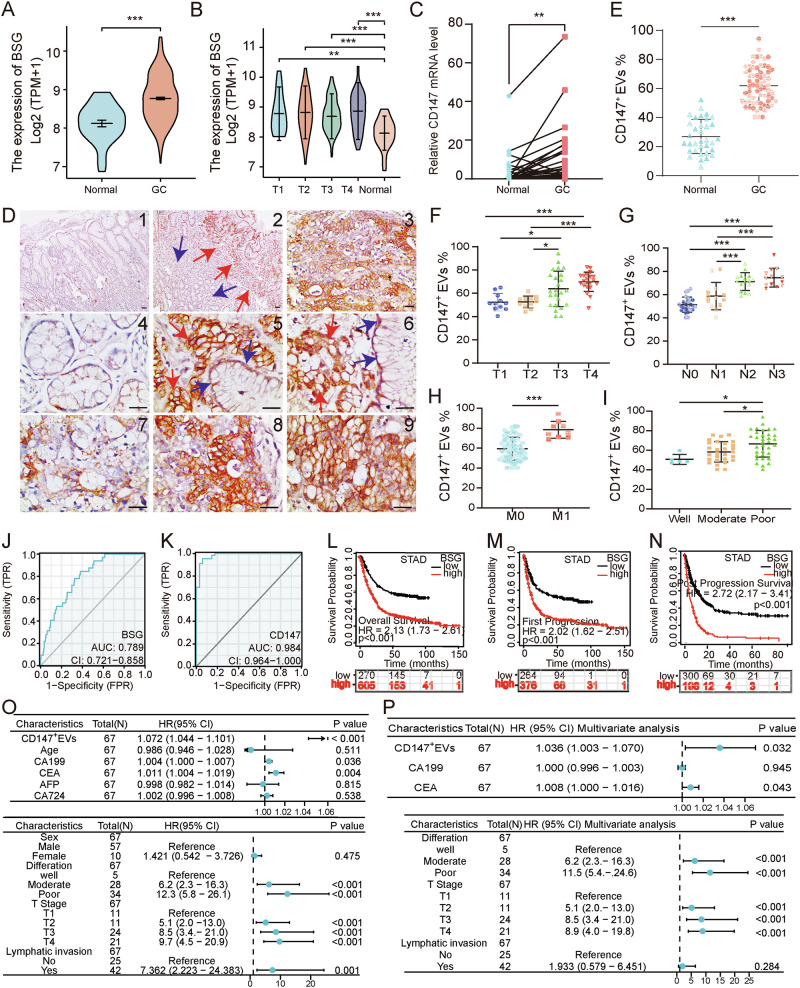
CD147 expression in gastric cancer patients demonstrates diagnostic potential and correlates with poor prognosis. **A** CD147 mRNA expression levels in gastric cancer (GC) tissues (*n* = 375) and normal tissues (*n* = 32) based on the TCGA database. **B** CD147 mRNA expression in GC patients at different T stages compared to normal tissues (TCGA database). **C** Relative CD147 mRNA expression in 53 GC tissues and paired adjacent normal tissues (*n* = 3, *t* test). **D** Immunohistochemical staining of CD147 in gastric cancer and adjacent normal tissues. (1, 4) Adjacent normal tissues, (2, 3, 5–9) Gastric cancer tissues (red arrows: cancerous tissues, blue arrows: normal mucosal glands adjacent to cancerous tissues, bar = 50 μm). **E** Flow cytometric analysis of CD147-positive EVs in plasma from GC patients (*n* = 67) and healthy subjects (*n* = 34). **F** CD147 expression across different T stages; **G** N stages; **H** M stages; and **I** differentiation grades. **J** ROC curve for CD147 mRNA expression in gastric tissues (TCGA database, GC *n* = 375, normal *n* = 32), AUC = 0.789. **K** ROC curve for CD147 expression in plasma EVs (GC patients *n* = 67, healthy subjects *n* = 34), AUC = 0.984. Kaplan–Meier analysis of CD147 expression and patient survival in GC patients. **L**–**N** Kaplan–Meier survival analysis of CD147 expression in GC patients: **L** overall survival (OS), **M** first progression (FP), and **N** post-progression survival (PPS). **O**, **P** Forest plots of univariate (**O**) and multivariate (**P**) Cox proportional hazards regression models. *p < 0.05, **p < 0.01, ***p < 0.001.

TCGA database was analyzed and revealed that the expression of *CD147* mRNA in GC tissues was significantly higher compared to normal tissues (Fig. 6A). Also, the expression of *CD147* mRNA in GC samples with different pathological stages was higher than that in normal tissues (Fig. 6B). Further, we collected cancer and para-cancer tissues of 53 patients with GC and confirmed that the expression of *CD147* mRNA in cancer tissues was markedly higher than that in para-cancer tissues (*p* < 0.01; Fig. 6C). The clinical correlation analysis illustrated that high expression of *CD147* mRNA was significantly associated with N stage (*p* = 0.021), lymph node metastasis (*p* = 0.008), and differentiation degrees (*p* = 0.035) (Supplementary Table S2). Immunohistochemistry staining verified that CD147 was mainly localized in the epithelial cell membranes, and the intensity was higher in cancer tissues than in para-cancer tissues (Fig. 6D).

In addition, we analyzed the expression of CD147 in plasma EVs from 67 GC patients and 34 healthy volunteers. The results exhibited that the positivity rate of CD147 in plasma EVs was significantly higher in GC patients than that in healthy controls (Fig. 6E), with notable differences across T stages (Fig. 6F), N stages (Fig. 6G), M stages (Fig. 6H), and varying differentiation levels in GC patients (Fig. 6I) (*p* < 0.05). To evaluate the clinical significance of CD147^+^ EVs in plasma, we stratified patients into high-CD147^+^ EVs and low-CD147^+^ EVs groups based on the median positivity rate of CD147 in plasma EVs. Elevated CD147^+^ EVs levels were significantly associated with advanced T stage, nodal metastasis, distant metastasis, higher pathological stage, increased lymphatic invasion, and poor differentiation(Table 1).

**Table 1:** Correlation between plasma extracellular vesicle CD147 expression and clinicopathological characteristics of gastric cancer patients.

Characteristic	Low expression of CD147	High expression of CD147	*p*
*n*	33	34	
T stage, *n* (%)			0.018
T1	2 (3%)	0 (0%)	
T2	10 (14.9%)	3 (4.5%)	
T3	16 (23.9%)	17 (25.4%)	
T4	5 (7.5%)	14 (20.9%)	
N stage, *n* (%)			<0.001
N0	22 (32.8%)	3 (4.5%)	
N1	9 (13.4%)	4 (6%)	
N2	2 (3%)	14 (20.9%)	
N3	0 (0%)	13 (19.4%)	
M stage, *n* (%)			0.005
M0	33 (49.3%)	25 (37.3%)	
M1	0 (0%)	9 (13.4%)	
Pathologic stage, *n* (%)			<0.001
IA	9 (13.4%)	1 (1.5%)	
IB	7 (10.4%)	1 (1.5%)	
IIA	9 (13.4%)	2 (3%)	
IIB	2 (3%)	2 (3%)	
IIIA	0 (0%)	1 (1.5%)	
IIIB	6 (9%)	20 (29.9%)	
IV	0 (0%)	7 (10.4%)	
Gender, *n* (%)			1.000
Female	5 (7.5%)	5 (7.5%)	
Male	28 (41.8%)	29 (43.3%)	
Lymphatic invasion, *n* (%)			<0.001
No	22 (32.8%)	3 (4.5%)	
Yes	11 (16.4%)	31 (46.3%)	
Differentiation, *n* (%)			0.003
Well	5 (7.5%)	0 (0%)	
Moderate	11 (16.4%)	4 (6%)	
Poor	17 (25.4%)	30 (44.8%)	
Age, mean ± SD	59.909 ± 8.6796	60.647 ± 9.5183	0.741

Receiver operating characteristic analysis demonstrated that the expression levels of *CD147* mRNA in gastric tissue (healthy = 32, GC = 375) from the TCGA database indicated moderate diagnostic accuracy for GC (AUC = 0.789) (Fig. 6J), while CD147 protein levels in plasma EVs (healthy = 34, GC = 67) exhibited higher diagnostic accuracy (AUC = 0.984, Fig. 6K). Kaplan–Meier analysis demonstrated that CD147-high gastric adenocarcinoma patients had significantly worse overall survival, faster disease progression, and reduced post-progression survival compared to CD147-low counterparts (all log-rank *p* < 0.001; Fig. 6L–N). Cox proportional hazards regression identified high CD147 expression in plasma EVs, lymph node metastasis, CA199, CEA, T stage, and differentiation status as independent predictors of poor prognosis in GC patients (*p* < 0.05; Fig. 6O). Multivariate analysis further confirmed that CD147 levels in plasma EVs, T stage, differentiation status, and CEA were significantly associated with poor prognosis (*p* < 0.05; Fig. 6P). High expression of CD147 in GC tissues and plasma EVs correlates with tumor progression and poor prognosis and shows promise as a diagnostic and prognostic biomarker for GC.

## Discussion

This study highlights the critical role of membrane proteins in the structural characteristics of gcEVs in modulating endothelial barrier function. Our results indicated that the enzymatic removal of these proteins significantly increased transendothelial resistance and reduced dextran permeability, suggesting that membrane proteins are essential for regulating endothelial permeability. Proteomic analysis identified CD147 (BSG) as a key upregulated protein in gcEVs, with GO analysis showing its involvement in processes such as cell morphology and focal adhesion. The association of CD147 with these processes underscores its potential role in modulating endothelial cell interactions and regulating endothelial permeability.

Previous studies demonstrate that CD147 overexpression correlates with aggressive clinicopathological features and poor prognosis in GC patients [24], primarily through its ability to upregulate matrix metalloproteinases MMP-2/MMP-9 [25], activate pro-angiogenic pathways via VEGF receptor cross-talk [26], and drive epithelial–mesenchymal transition through STAT3/AKT/ERK signaling [27–29]. While our study uncovers a novel EV-mediated paradigm of CD147’s endothelial modulation. Specifically, we provide the first evidence that CD147-high gcEVs significantly enhanced vascular permeability, promoting tumor cell transendothelial migration. After the injection of CD147-high gcEV into the tail vein of mice, the vascular permeability of pulmonary was significantly increased, promoting tumor cell metastasis to the lung and liver. These findings suggest that CD147-high gcEVs modulate endothelial barrier function to create a permissive environment for tumor cell invasion and metastasis. This aligns with previous reports that tumor-derived EVs play a crucial role in preparing the pre-metastatic niche by increasing vascular permeability [30, 31]. Interestingly, while recombinant CD147 protein increased vascular permeability, its effects were less pronounced than those of CD147-high gcEVs. This difference may be attributed to the synergistic actions of other proteins and RNA present in EVs [32], which collectively modulate endothelial cell function. Additionally, the CD147 carried by EVs can be efficiently internalized by endothelial cells, facilitating more effective signal transduction and regulation of endothelial cell activity.

Moreover, the uptake of CD147-high gcEVs by endothelial cells induced cytoskeletal rearrangements, which disrupted the orderly endothelial structure. Cytoskeletal disorganization is a critical step in endothelial dysfunction, leading to increased vascular permeability. Furthermore, VE-cadherin, a key regulator of endothelial cell junction integrity [23, 33, 34], was internalized following treatment with CD147-high gcEVs, further compromising the endothelial barrier. Previous studies have demonstrated that endothelial cytoskeletal rearrangements and VE-cadherin internalization led to barrier disruption [35–37], supporting our findings that CD147-high gcEVs initiate endothelial dysfunction. These results underscore the critical role of CD147-high gcEVs in reshaping the endothelial microenvironment to promote tumor metastasis.

Additionally, our study highlighted the importance of CD147-high EVs in modulating endothelial cell function through the VEGF/AKT/eNOS/NO and AKT/mTOR/p70S6K signaling pathways. These two pathways are known to regulate vascular permeability and angiogenesis [38–40], as well as cell adhesion [41, 42]. As a major downstream effector of AKT, mTOR is closely related to the biological behavior of malignant neoplasms [43]. We found that the expression of CD147 was positively correlated with the activation of these pathways, highlighting their involvement in endothelial cell modulation. Inhibition of the AKT or mTOR pathway further validated its pivotal role in the regulation of endothelial cell function by CD147-high gcEVs and its impact on GC metastasis. While previous studies have shown that EVs from various cancers modulate the TME by delivering oncogenic proteins and RNA to recipient cells [44, 45], our research uniquely elucidates the specific mechanisms through which CD147-high EVs disrupt the endothelial barrier and facilitate tumor cell dissemination in GC.

Importantly, we assessed the clinical relevance of CD147 as a diagnostic and prognostic biomarker for GC. Previous studies have demonstrated the diagnostic relevance of EVs in multiple cancers [46–48]. Our analysis revealed that high CD147 expression in GC tissues and patient plasma was strongly associated with poor clinical outcomes. Notably, CD147 positivity in plasma EVs was significantly higher in GC patients than in healthy controls, demonstrating its utility for non-invasive diagnostics. The exceptional diagnostic accuracy of plasma CD147^+^ EVs (AUC = 0.984) surpasses traditional tissue-based markers (AUC = 0.789 for CD147 mRNA), highlighting its potential for early detection and liquid biopsy applications. Additionally, elevated CD147 expression was linked to shorter survival times, establishing it as an independent predictor of poor prognosis alongside traditional markers like CA199 and CEA. Multivariate analysis reinforced the critical role of CD147 levels in plasma EVs for prognostic assessments, underscoring its clinical relevance in GC management.

In conclusion, this study reveals that CD147-high EVs derived from GC play a critical role in modulating endothelial cell function, disrupting the vascular barrier, and promoting tumor metastasis via the VEGF/AKT/eNOS/NO and AKT/mTOR/p70S6K signaling pathways. These findings offer new insights into the molecular mechanisms underlying GC invasion and metastasis and highlight the potential clinical application of CD147-high EVs as diagnostic and prognostic biomarkers. Future research should further explore the mechanisms by which CD147-high gcEVs contribute to tumor progression and investigate strategies to block their effects, with the aim of developing more effective therapeutic approaches for GC patients.

## Supplementary information

SUPPLEMENTAL MATERIALS AND METHODS

## Data Availability

Only publicly available data were used in this study. The public database can be found through URL as listed in the article. Other original data in this study are available from the corresponding author upon request and approval of a research proposal.
