# Supplementary material for: CD147-high extracellular vesicles promote gastric cancer metastasis via VEGF/AKT/eNOS and AKT/mTOR pathways
Source: Oncogenesis. 2025 Jun 20;14(1):21. doi: 10.1038/s41389-025-00564-3 (PMC12181333; doi:10.1038/s41389-025-00564-3)
Supplement: Supplementary file 1 — SUPPLEMENTAL MATERIALS AND METHODS [file 41389_2025_564_MOESM1_ESM.docx]

**Supplemental** **materials and methods**

**Transmission Electron Microscopy (TEM)**

Ultrathin Sectioning Method. The gcEVs pellet was fixed with 2.5% glutaraldehyde at 4°C for 24 hours. After washing with PBS three times, the samples were fixed again with 1% osmium tetroxide for 1.5 hours at 4°C, washed again three times, and dehydrated with graded ethanol (50%, 70%, 80%, 90%, 100%)for 10 minutes at each step. They were then infiltrated with Epon 812 for 3 hours and polymerized at 37°C, 45°C and 60°C for 24 hours, respectively. Ultrathin sections (50 nm) were sliced using an ultramicrotome, stained with uranyl acetate and lead citrate, and observed under a transmission electron microscope (TEM; JEM-1230, JEOL, Japan) to confirm the presence of round, double-layered EVs.

Negative Staining with Phosphotungstic Acid. EV suspensions (untreated, 10% trypsin-treated, or 10% SDS-treated) were placed on Formvar-coated copper grids (25 μL per sample) and incubated for 2 minutes at RT. Excess liquid was removed with filter paper, and grids were stained with 20 μL of 120 mmol/L phosphotungstic acid (pH 6.8) for 2 minutes. After staining, remaining liquid was absorbed, and grids were air-dried under an incandescent lamp. The samples were then observed and imaged using a transmission electron microscope.

**Nanoparticle Tracking Analysis (NTA)**

Nanoparticle tracking analysis (NTA) was performed as previously described[1, 2]. Briefly, 50μL gcEVs were diluted in an appropriate volume of buffer, and the particle concentration and size distribution were analyzed using a NanoSight NS300 detection system (Malvern Instruments, Great Malvern, UK).

**Flow Cytometry Analysis of Extracellular Vesicles**

All buffers (phosphate‐buffered saline , staining buffer were pre‐filtered through 0.1 μm filters (Millipore, USA) to remove particulate contaminants. EVs were analyzed following our previously established protocols with modifications to optimize plasma EVs detection[3, 4].

Gating Strategy:Standard microspheres (Spherotech, USA) with diameters of 0.22, 0.88, and 3 μm were used to calibrate the instrument and define the gating strategy. The EV population was defined as particles with a size of <1 μm based on the bead calibration.

For GC cell-Derived EVs:EVs isolated from GC cell culture supernatants were stained with FITC-Annexin V (BD Biosciences, USA) to label phosphatidylserine on the EV membranes and with PerCP/Cy5.5-conjugated anti-human CD326 (EpCAM; BioLegend, USA) to identify EVs of epithelial (tumor) origin. Appropriate isotype controls were used to verify antibody specificity.

For Plasma EVs Plasma samples collected from patients and healthy volunteers were centrifuged at 150×g for 20 min at RT to obtain platelet-rich plasma, which was centrifuged at 1,500×g for 20 min to collect platelet-poor plasma (PPP). PPP was centrifuged at 13,000×g for 2 min at 4°C to collect the cell-free plasma.100 μL of cell-free plasma was incubated with 5 μL of FITC-Annexin V and 5 μL of rabbit anti-human APC-CD147 antibody (BioLegend, USA) for 30 minutes at room temperature in the dark.Negative controls (unstained samples and isotype control antibodies) and single-stained controls were prepared for proper compensation.The instrument (BD FACSCanto™ II, Becton Dickinson, USA) was calibrated using the standard microspheres, and the EV population was gated based on the size (<1 μm). FITC-Annexin V was used to confirm the presence of membrane vesicles, and the percentage of APC-CD147-positive EVs was quantified.

**Lentiviral-Mediated CD147 Silencing**

HGC-27 and AGS cells were seeded in 6-well plates (5×10^4^ cells/well) and cultured to 30% confluence. The cells were then infected with GV248-CD147-shRNA (Shanghai Genechem Co., Ltd.) or negative control shRNA at a multiplicity of infection (MOI) of 30. After 96 hours, infection efficiency was determined by counting GFP-expressing cells under a fluorescence microscope (ECLIPSE 80i, Nikon, Japan).

**Quantitative Real-Time PCR (qRT-PCR)**

Tissue samples: A total of 53 paired gastric cancer tissues and adjacent normal tissues were collected under ethical approval.Total RNA from cells(HGC-27,AGS) and tissues (53 paired gastric cancer tissues) was extracted using Trizol reagent (Invitrogen, USA). cDNA was synthesized using M-MLV reverse transcriptase (Promega, USA) and random hexamers (Takara, Japan). RT-PCR was performed using QuantStudio 3 (ABI, USA) and Hieff UNICON® qPCR SYBR Green Master Mix (Yeasen Biotechnology, China). Primer sequences were as follows: CD147, forward 5′-AGTGTAGCCACATTCCTGCC-3′, reverse 5′-GGCGTCTCTTGGAGGTTGAA-3′; GAPDH, forward 5′-ACAGTCAGCCGCATCTTCTT-3′, reverse 5′-GGATGCCACAGGACTCCAT-3′. The thermocycling conditions were set as: 95˚C for 2 minutes (hold stage); 95˚C for 10 sec, 60˚C for 30 sec (40 cycles, PCR stage); 95˚C for 15 sec, 60˚C for 1 minute, 95˚C for 1 sec,25˚C for 5 minutes (melt curve stage). The relative fold changes of mRNA were calculated using the comparative cycle threshold (2^-ΔΔCq^) method[5]. The assay was repeated three times.

**Western Blot**

Total protein was extracted from cells and EVs, and quantified using the BCA protein assay (Solarbio Science & Technology Co., Ltd., Beijing, China). The protein samples were separated by 8% SDS-PAGE and transferred to PVDF membranes (Millipore, Billerica, MA). After blocked with 5% BSA, the membranes were incubated overnight at 4°C with primary antibodies: CD147 (1:1000, Abcam,ab108308,Cambridge, MA, USA) , VEGF (1:1000, Gene Tex,GXT102643), p-AKT (1:1000, CST,9271,Danvers, MA, USA), AKT (1:1000, CST,9272), p-mTOR (1:1000, Gene Tex,GTX133190), mTOR (1:1000, Gene Tex,GXT101557), p-p70S6K (1:1000, CST,9234), p70S6K (1:1000, CST,34475) ,p-eNOS (1:1000, Gene Tex,GTX129058), GAPDH (1:5000, Immunoway,YM3215，Plano, TX) , and β-tubulin (1:5000, Immunoway, YT4780). Membranes were then incubated secondary horseradish peroxidase (HRP)-labeled antibodies (goat anti-rabbit IgG or goat anti-mouse IgG) for 2 hours at RT and visualized using Super ECL Detection Reagent (Yeasen Biotechnology, China). The changes in the abundance of proteins were determined by densitometric analysis using ImageJ Software 1.4.3.67 (National Institutes of Health, Bethesda, MD, USA) and normalized to GAPDH or β-tubulin.

**Tissue Histology**

Mice were euthanized under anesthesia, and organs were dissected, rinsed with cold PBS, and fixed in 4% paraformaldehyde. Tissues were dehydrated, paraffin-embedded, sectioned, and stained with H&E. Sections were scanned using the Pannoramic DESK system (3DHISTECH, Hungary), and images were analyzed with Pannoramic Case Viewer 2.4. Quantitative analysis was performed using Quant Center 2.1. Tumor burden (%) was calculated as the tumor area relative to lung or liver cross-sectional area (tumor burden = [tumor area / lung or liver area] × 100%).

**Immunohistochemical Staining**

CD147 expression in gastric cancer and adjacent tissues was assessed using the SP-9001 kit (Beijing Zhongshan Golden Bridge Biotechnology Co., China). Tissue samples were fixed in 10% formalin, dehydrated, and sectioned to 5 μm. Sections were deparaffinized, rehydrated, and subjected to antigen retrieval. After blocking with goat serum, sections were incubated overnight with primary antibody (1:200), followed by a biotinylated secondary antibody (1:200) and horseradish peroxidase-labeled streptavidin. DAB solution was applied for color development, and sections were counterstained with hematoxylin. Two pathologists independently scored the staining in five high-power fields, using a semi-quantitative scale based on staining intensity and percentage of positive cells.

**Nitric Oxide (NO) Measurement**

HUVECs were cultured in 24-well plates until 70-80% confluence. The medium was replaced, and different EVs (Mock, shNC, shCD147) at 5000 particles/μL, PBS, or MK2206 (20 μM) were added. Cells were incubated for 24 hours at 37°C in a 5% CO2 incubator. After incubation, 100 μL of culture supernatant from each well was collected. Reagent 1 (200 μL) from the NO detection kit was added and mixed thoroughly, followed by Reagent 2 (100 μL). The mixture was vortexed and left to stand for 10 minutes, then centrifuged at 3500 rpm for 15 minutes. From the supernatant, 160 μL was collected, and the assay was performed according to the kit's instructions.The liquid was mixed and left to stand for 15 minutes. The optical density (OD) at 550 nm was measured using a microplate reader. NO concentration was calculated using the following formula: NO concentration (μmol/L) = (A sample – A blank) / (A standard – A blank) × C standard × dilution factor.

**Tumor markers**

Blood samples were collected pre-surgery to measure four common tumor markers: carcinoembryonic antigen (CEA; Roche Diagnostics GmbH, Mannheim,Germany), carbohydrate antigen 199 (CA199; Roche), alpha-fetoprotein (AFP; Roche) and carbohydrate antigen 724 (CA724; Roche). Measurements were conducted using a chemiluminescence microparticle immunoassay on the cobas 8000 e 801 automatic immunoassay analyzer (Roche, Germany).

**Bioinformatics analysis**

Bioinformatics analysis was performed using RNA-seq data in HTSeq-FPKM format and clinical data from the TCGA database (https://portal.gdc.cancer.gov/) for the STAD (stomach cancer) project to evaluate CD147 expression. Statistical analysis and visualization were conducted in R (version 3.6.3), primarily using the ggplot2 package (version 3.3.3). FPKM data were converted to TPM (Transcripts Per Million) format and log2-transformed. Pathway enrichment was analyzed using the Kyoto Encyclopedia of Genes and Genomes (KEGG). Protein interaction analyses were conducted using the STRING database (http://string-db.org) and STITCH database (http://stitch.embl.de). The association between CD147 expression and gastric cancer prognosis (overall survival, disease-free survival, and post-progression survival) was assessed using Kaplan-Meier analysis and log-rank tests, with the cutoff set at the median expression level of CD147.

**References**

1.Shao H, Im H, Castro CM, Breakefield X, Weissleder R, Lee H. New Technologies for Analysis of Extracellular Vesicles. Chem Rev 2018; 118: 1917-1950.

2.Van Der Pol E, Coumans FA, Grootemaat AE, Gardiner C, Sargent IL, Harrison P et al. Particle size distribution of exosomes and microvesicles determined by transmission electron microscopy, flow cytometry, nanoparticle tracking analysis, and resistive pulse sensing. J Thromb Haemost 2014; 12: 1182-1192.

3.Wang M, Cai W, Yang AJ, Wang CY, Zhang CL, Liu W et al. Gastric cancer cell-derived extracellular vesicles disrupt endothelial integrity and promote metastasis. Cancer Lett 2022; 545: 215827.

4.Went P, Vasei M, Bubendorf L, Terracciano L, Tornillo L, Riede U et al. Frequent high-level expression of the immunotherapeutic target Ep-CAM in colon, stomach, prostate and lung cancers. Br J Cancer 2006; 94: 128-135.

5.Ma H, Bell KN, Loker RN. qPCR and qRT-PCR analysis: Regulatory points to consider when conducting biodistribution and vector shedding studies. Molecular Therapy - Methods & Clinical Development 2021; 20: 152-168.

**Supplemental TABLES**

**Supplementary Table 1. Summary of Mass Spectrometry Data**

| **Total spectrum** | **Matched spectrum** | **Peptides** | **Unique peptides** | **Identified proteins** | **Quantifiable proteins** |
| --- | --- | --- | --- | --- | --- |
| **1003552.0** | **76609** | **26282.0** | **25111.0** | **5001.0** | **2185.0** |

**Supplementary Table 2. Correlation between CD147 mRNA expression and clinicopathological characteristics in gastric cancer tissues.**

|  | Low expression of BSG | Hight expression of BSG | *p* |
| --- | --- | --- | --- |
| n | 26 | 27 |  |
| T stage, n (%) |  |  | 0.929 |
| T1 | 2 (3.8%) | 3 (5.7%) |  |
| T2 | 3 (5.7%) | 4 (7.5%) |  |
| T3 | 10 (18.9%) | 8 (15.1%) |  |
| T4 | 11 (20.8%) | 12 (22.6%) |  |
| N stage, n (%) |  |  | 0.021 |
| N0 | 15 (28.3%) | 5 (9.4%) |  |
| N1 | 2 (3.8%) | 2 (3.8%) |  |
| N2 | 5 (9.4%) | 11 (20.8%) |  |
| N3 | 4 (7.5%) | 9 (17%) |  |
| M stage, n (%) |  |  | 1.000 |
| M0 | 26 (49.1%) | 26 (49.1%) |  |
| M1 | 0 (0%) | 1 (1.9%) |  |
| Pathologic stage, n (%) |  |  | 0.350 |
| IA | 2 (3.8%) | 2 (3.8%) |  |
| IB | 1 (1.9%) | 2 (3.8%) |  |
| IIA | 6 (11.3%) | 3 (5.7%) |  |
| IIB | 4 (7.5%) | 1 (1.9%) |  |
| IIIA | 6 (11.3%) | 5 (9.4%) |  |
| IIIB | 5 (9.4%) | 5 (9.4%) |  |
| IIIC | 2 (3.8%) | 8 (15.1%) |  |
| IV | 0 (0%) | 1 (1.9%) |  |
| Gender, n (%) |  |  | 0.486 |
| female | 9 (17%) | 6 (11.3%) |  |
| male | 17 (32.1%) | 21 (39.6%) |  |
| Lymph node involvement,n (%) |  |  | 0.008 |
| No | 15 (28.3%) | 5 (9.4%) |  |
| Yes | 11 (20.8%) | 22 (41.5%) |  |
| Differation, n (%) |  |  | 0.035 |
| Moderate | 9 (17%) | 2 (3.8%) |  |
| Poor | 17 (32.1%) | 25 (47.2%) |  |
| Age, mean ± SD | 64.23 ± 11.87 | 61.93 ± 10.37 | 0.455 |


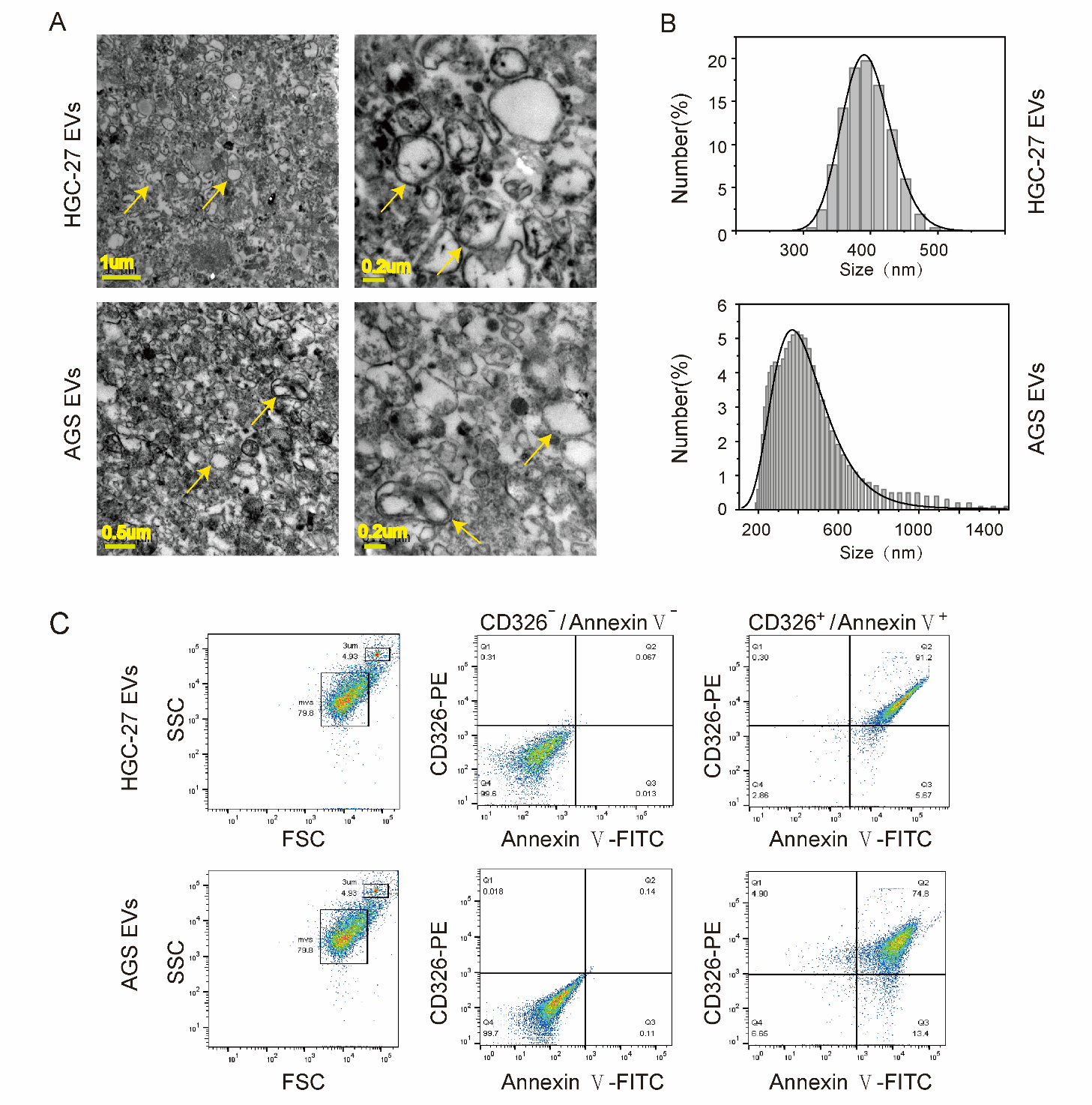
**Supplemental Figures and legends**

Supplementary Fig. S1. Characterization of extracellular vesicle (EVs) derived from HGC-27 and AGS cells. (A) Transmission electron microscopy (TEM) images showed the morphology of EVs. (B) Nanoparticle tracking analysis (NTA) depicted the size and particle distribution of EVs. (C) Flow cytometry was used to detect markers of gcEVs.


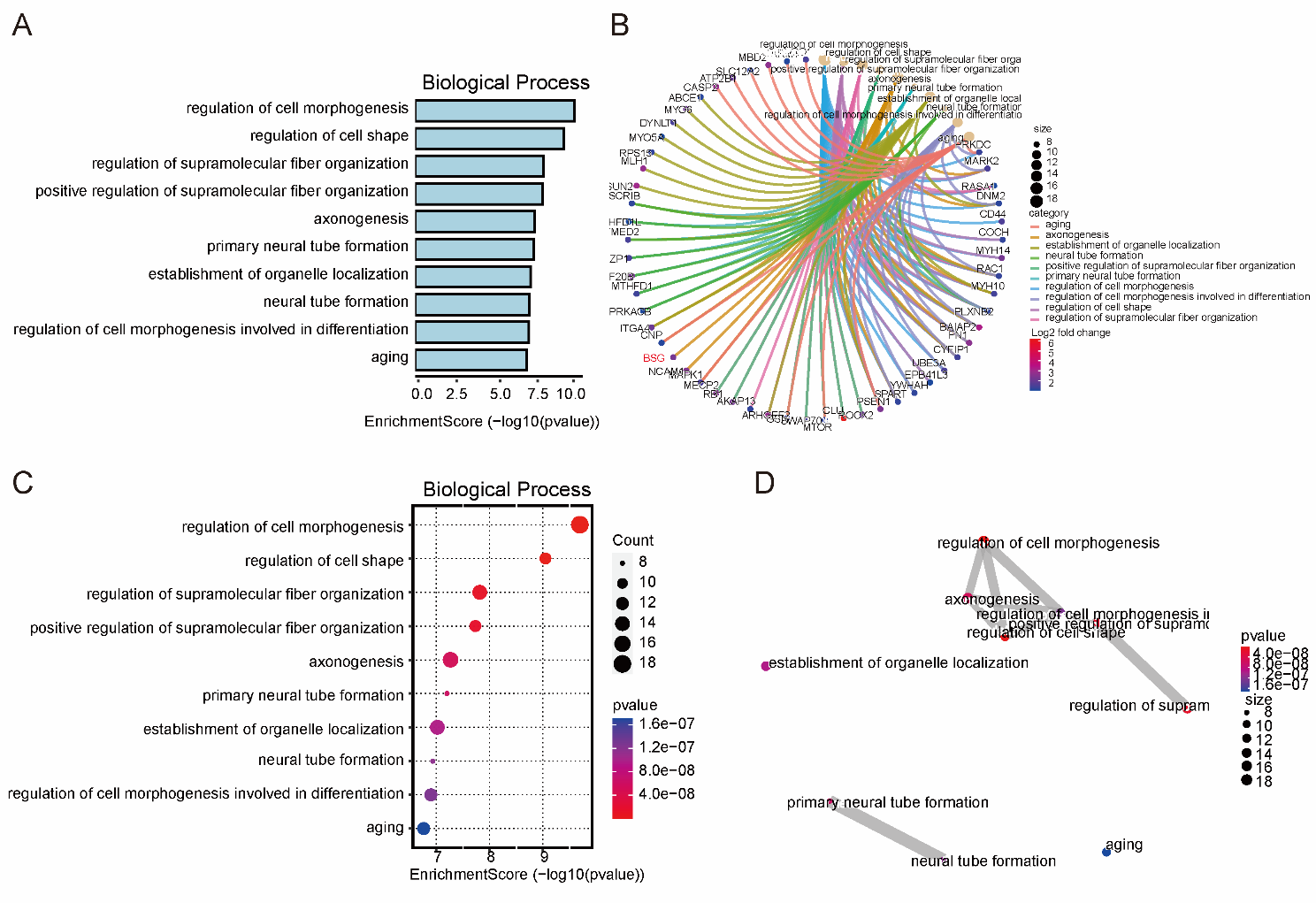
Supplementary Fig. S2. Biological process (BP) analysis of upregulated proteins in HGC-27 EVs. (A) Top ten biological processes enriched with upregulated proteins in HGC-27 EVs. (B) Network map illustrating the relationship between upregulated proteins and their associated BP. (C) Enrichment analysis of biological processes linked to upregulated proteins. (D). Interaction network of top 10 upregulated BP terms. Node size corresponds to the number of enriched proteins (larger circles indicate more proteins) .Node color indicates the significance level (-log10 transformed p-value, darker red indicates greater significance). Gray edges represent functional relationships, with edge width proportional to shared gene counts. The network was constructed using Cytoscape (v3.9.1), with FDR < 0.05 as the significance threshold.
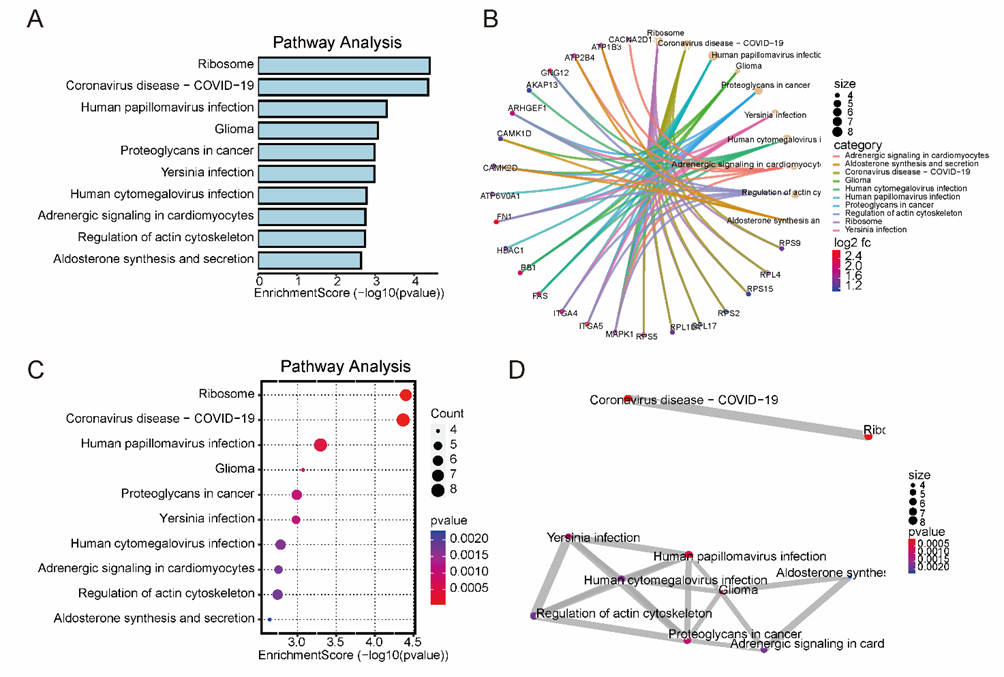
Supplementary Fig. S3. KEGG pathway enrichment analysis of GO molecular function-upregulated proteins in HGC-27-derived EVs (Fold change > 2, *p* < 0.05) (A) Bar plot of top 10 enriched KEGG pathways ranked by significance (-log10(p-value)). (B) Protein-KEGG pathway association network. (C) Bubble chart illustrating the enrichment of these KEGG pathways, with bubble size indicating the number of proteins and color reflecting the significance level (D) Interaction network of the top 10 enriched KEGG pathways. In this network, node size corresponds to the number of enriched proteins (larger circles indicate more proteins), and node color indicates the significance level (darker red indicates greater significance). Gray edges represent functional relationships between pathways, with edge width proportional to the number of shared genes. The network was constructed using Cytoscape (v3.9.1) with an FDR < 0.05 as the significance threshold.

Supplementary
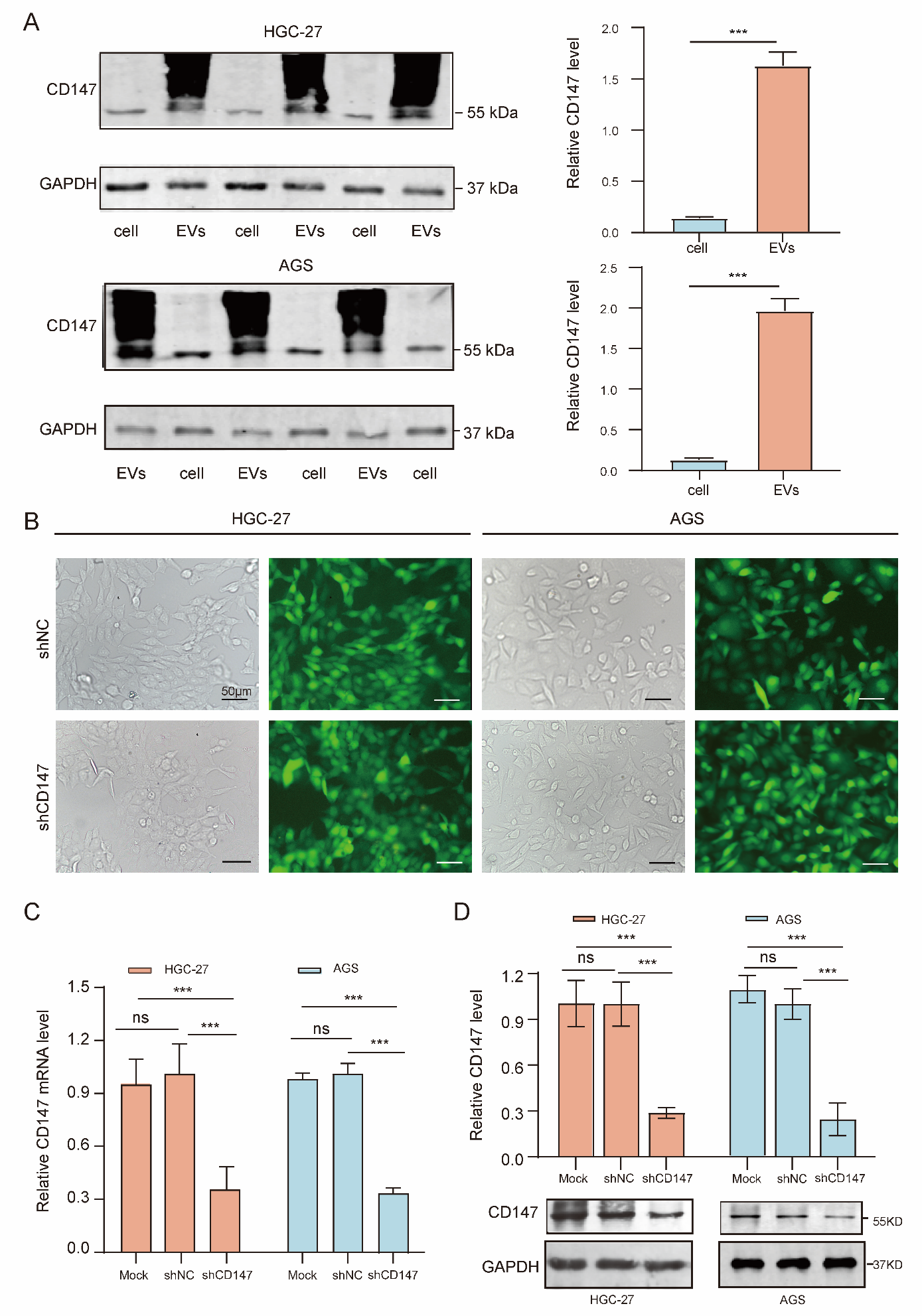
Fig. S4. Validation of CD147 expression. (A) Western blot analysis of CD147 expression in HGC-27 and AGS cells and their EVs; right panel shows statistical analysis (n = 3, t-test). (B) Construction of CD147 knockdown lentiviral vectors and their transfection into HGC-27 and AGS cells. The infection efficiency was observed under a fluorescence inverted microscope. The right panel showed fluorescent images of infected cells, and the left panel showed corresponding bright-field images (Bar = 50 μm). (C) qPCR analysis of CD147 mRNA expression in knockdown cells (n = 3, one-way ANOVA). (D) Western blot analysis of CD147 protein expression levels in knockdown cells, with corresponding quantification in the bar graph (n = 3, one-way ANOVA). ns = not statistically significant, **p* < 0.05, ***p* < 0.01, ****p* < 0.001.

Supplementary
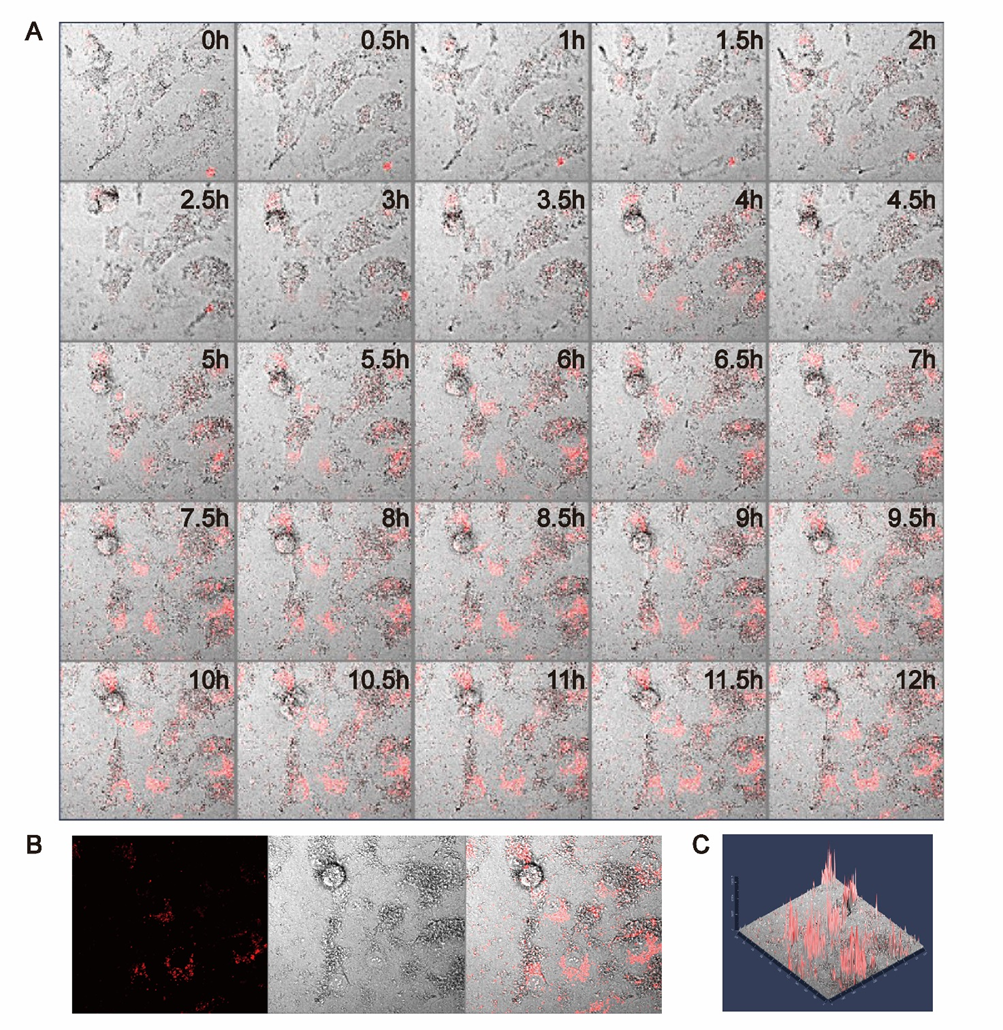
Fig.S5. Uptake of CD147-high extracellular vesicles by HUVEC. (A) Time-lapse imaging of PKH-26-labeled EVs (red) co-cultured with HUVECs for 12 hours, which was captured using live-cell imaging workstation. Images were taken every 0.5 hours in the same field of view. (B) Confocal microscopy image of HUVECs co-culture with labeled EVs for 12 hours (Bar = 20μm). (C) 3D reconstruction of the 12-hour time point showing the uptake of labeled EVs by HUVECs.


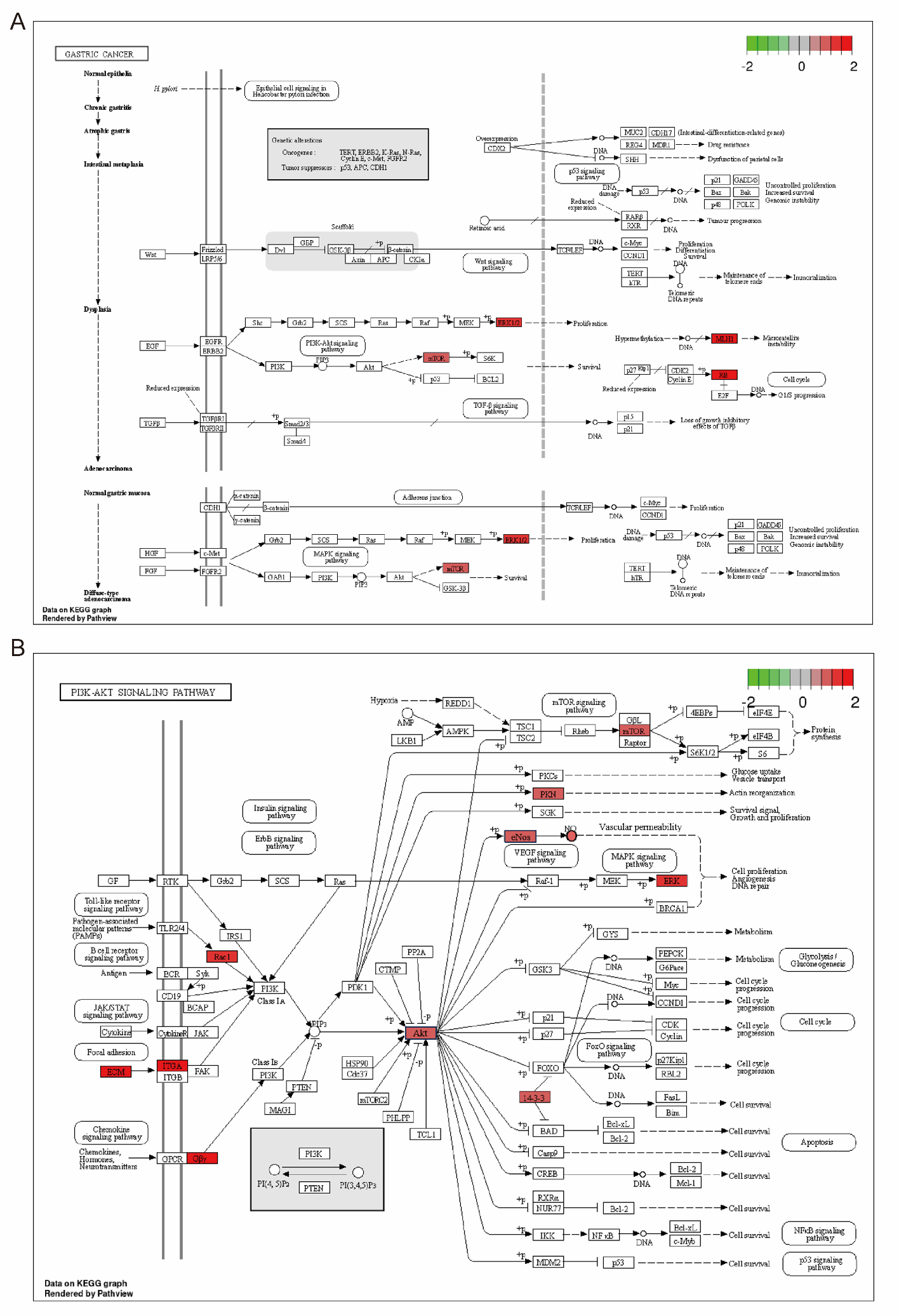


Supplementary Fig. S6. KEGG pathway mapping of MF-upregulated proteins in HGC-27-derived EVs(Fold change > 2, *p* < 0.05; Mapped via KEGG Mapper, https://www.kegg.jp/kegg/mapper.html) (A) Gastric cancer pathway (KEGG map:hsa05226) Proteins mapped to this pathway are highlighted in red, including mTOR, S6K, ERK1/2, MLH1, and RB.(B) PI3K-AKT signaling pathway (KEGG map:hsa04151). Proteins mapped to this pathway are highlighted in red, including AKT, eNOS , mTOR and S6K1/2.

Supplementary
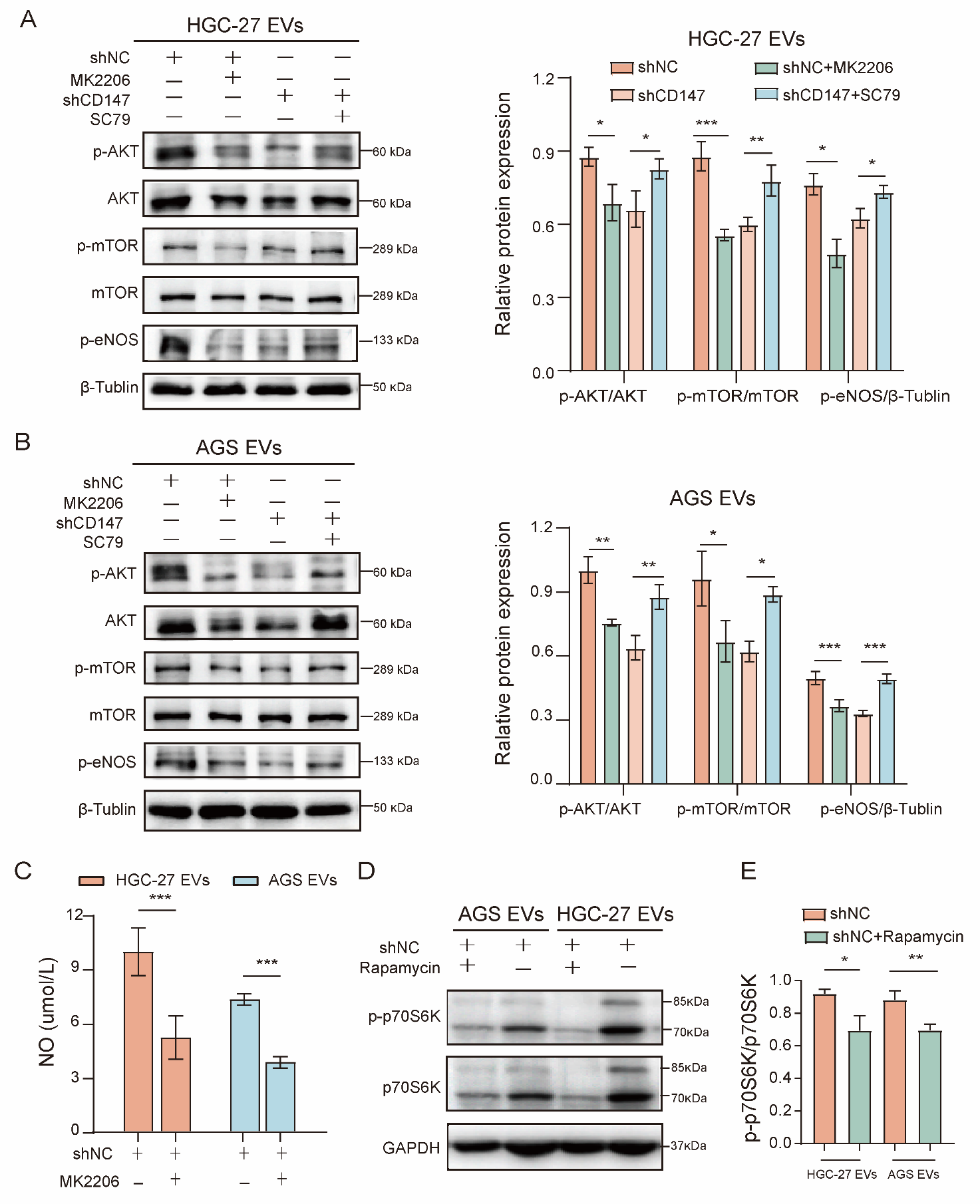
 Fig. S7. Effects of MK2206 and SC79 on protein expression in HUVECs treated with different EVs.Western blotting detected p-AKT, AKT, p-eNOS, p-mTOR, and mTOR in HUVECs treated with EVs derived from (A) HGC-27 cells and (B) AGS cells. The bar graphs represent the quantification of band intensities (n = 3, t-test). (C) Effect of MK2206 on nitric oxide (NO) release in HUVECs (n = 3, t-test). (D)Effects of Rapamycin on protein expression in HUVECs treated with EVs . Western blotting detected p-p70S6K and p70S6K. (E) Quantitative statistical analysis(n = 3, t-test)
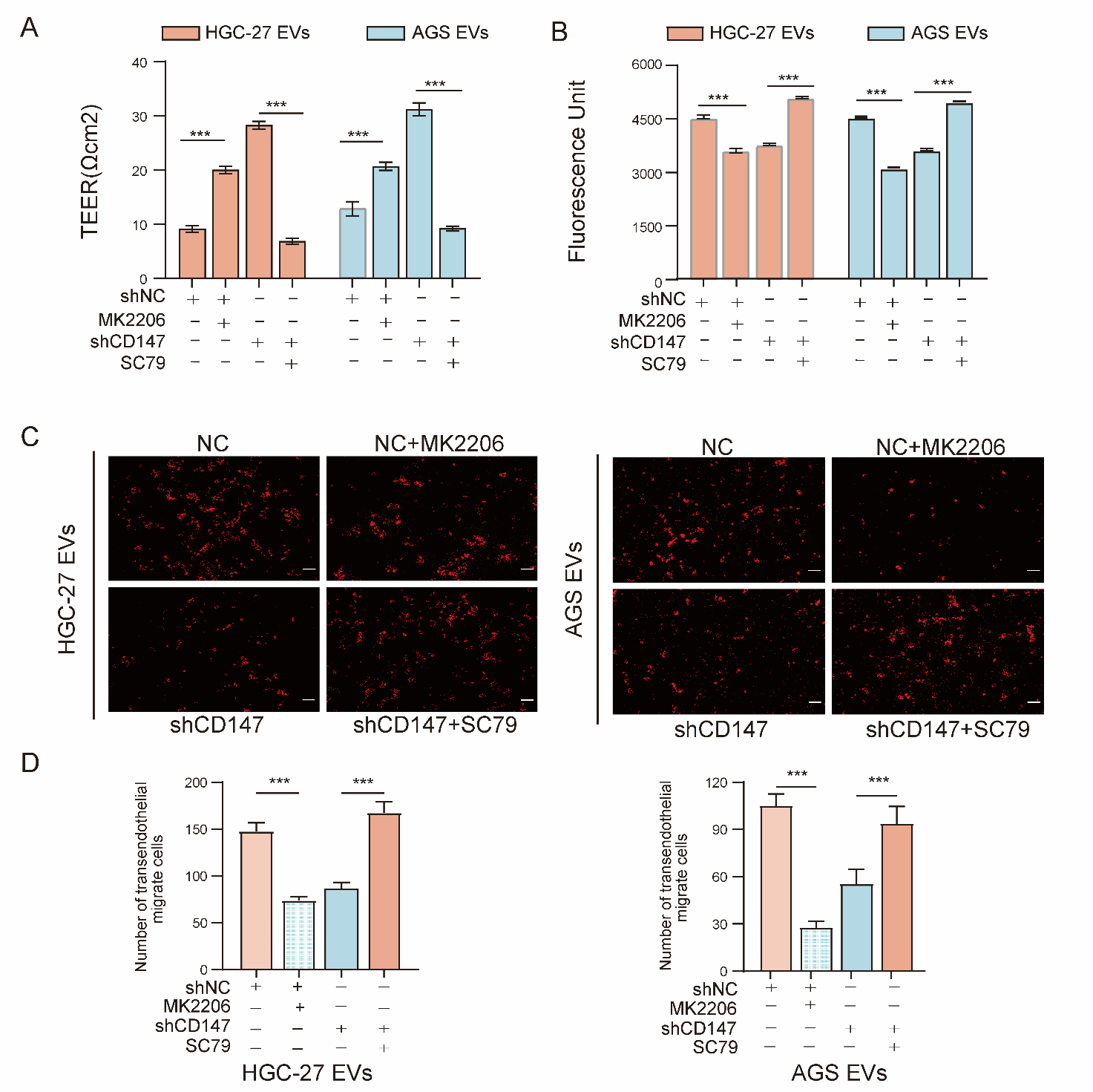
**p*
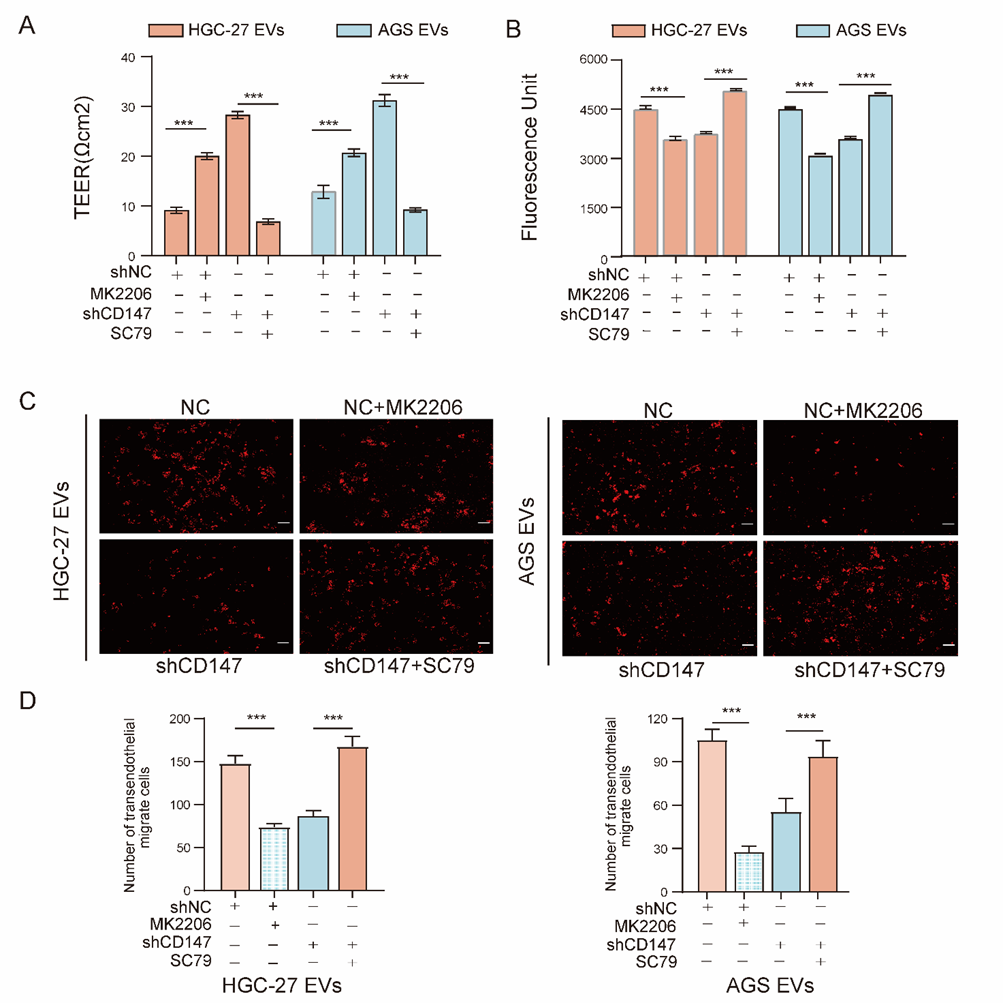
< 0.05, ***p* < 0.01, ****p* < 0.001.

Supplementary Fig. S8. CD147-high gcEVs regulated HUVEC permeability and transendothelial migration of tumor cells via the AKT pathway. HUVECs were treated with EVs from different experimental groups for 24 hours. (A) Trans-endothelial electrical resistance (TEER) measurements (n=3, t-test). (B) FITC-Dextran permeability assay, showing fluorescence intensity (n=3, t-test). (C) Transendothelial migration of tumor cells, with PKH-26-labeled HGC-27 cells (red fluorescence) visualized under a fluorescence microscope; quantification of migrated tumor cells was performed using ImageJ (Bar = 100 μm). (D) Bar graph quantified tumor cell transendothelial migration (n=3, t-test). **p*< 0.05, ***p*< 0.01, ****p*< 0.001.


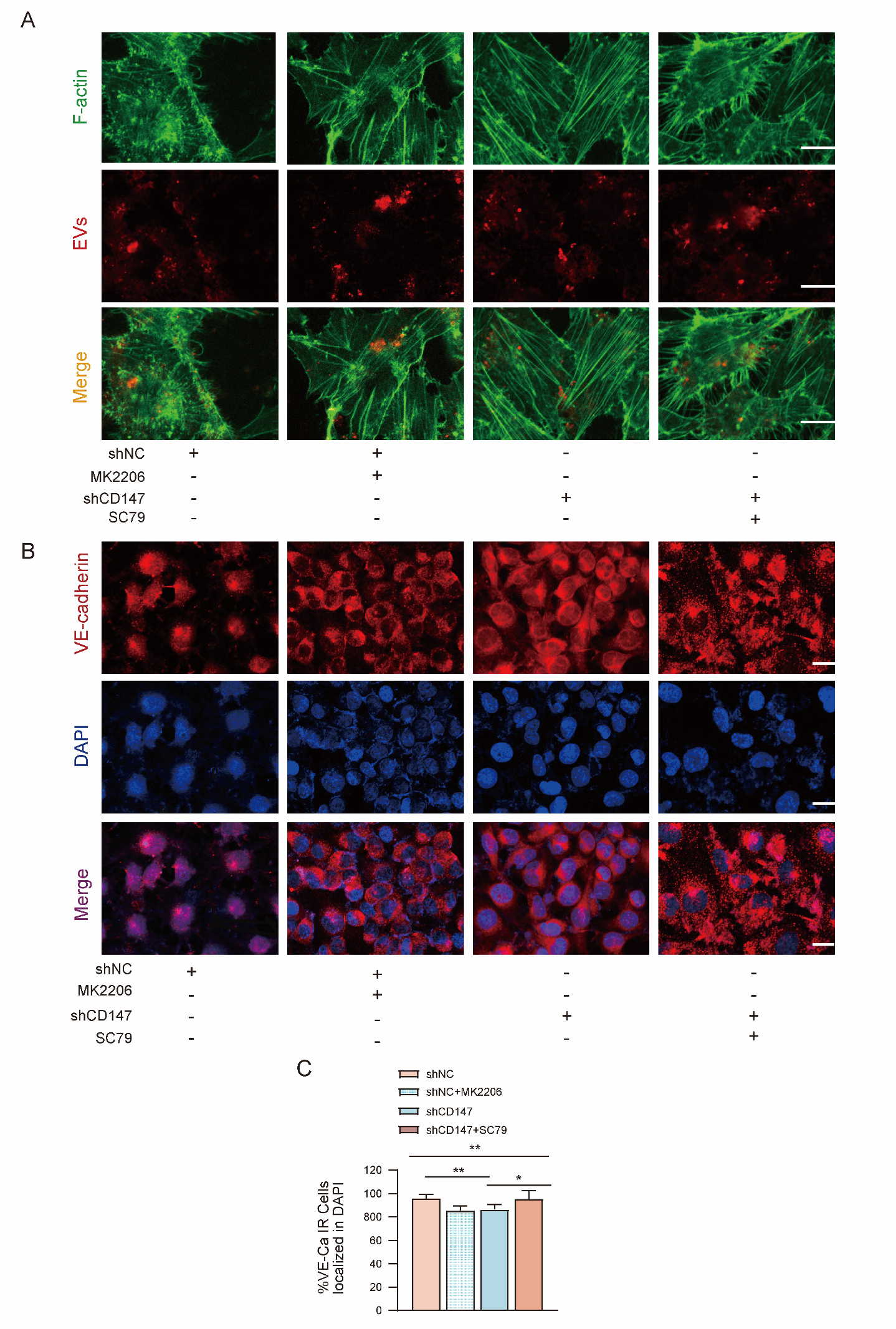


Supplementary Fig. S9. CD147-high gcEVs regulated cytoskeletal rearrangement and VE-cadherin localization in HUVEC via the AKT pathway. HUVECs were treated with EVs from different experimental groups for 24 hours. (A) Confocal laser scanning microscopy of endothelial cells showing F-actin distribution after 24-hour treatment (green: F-actin; red: EVs; Bar = 20 μm). (B) VE-cadherin expression and localization (red: VE-cadherin; blue: nucleus; Bar = 20 μm). (C) The colocalization coefficient of nuclear VE-cadherin shown (n=3, One-way ANOVA). **p*< 0.05, ***p*< 0.01, ****p*< 0.001.


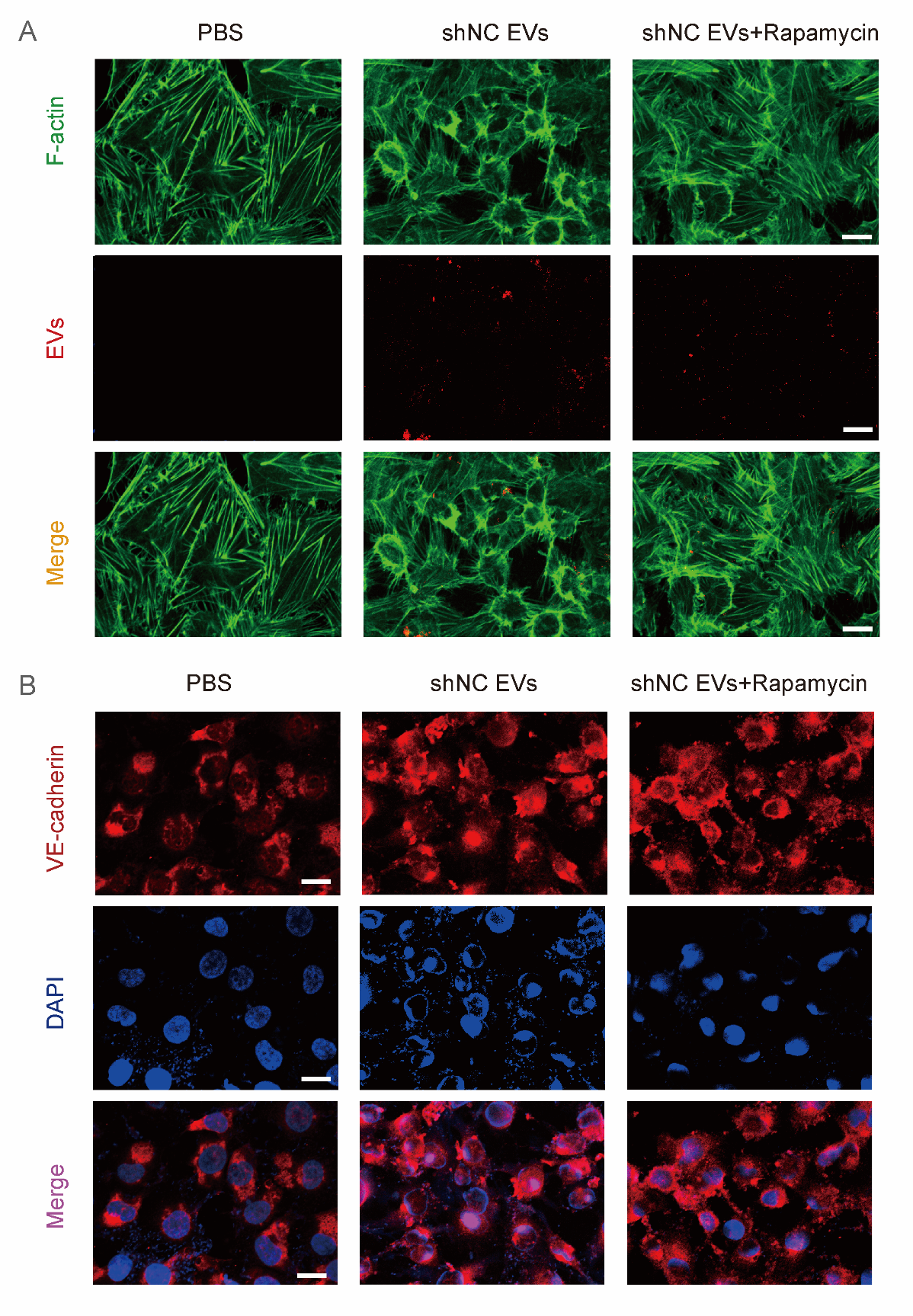
Supplementary Fig. S10. CD147-high gcEVs regulated cytoskeletal rearrangement and VE-cadherin localization in HUVEC via the mTOR pathway. HUVECs were treated with EVs from different experimental groups for 24 hours. (A) Confocal laser scanning microscopy of endothelial cells showing F-actin distribution after 24-hour treatment (green: F-actin; red: EVs; Bar = 20 μm). (B) VE-cadherin expression and localization (red: VE-cadherin; blue: nucleus; Bar = 20 μm).
